# Potential Risk of Cutaneous Melanoma Attributable to Medication Use: A Mendelian Randomization Approach

**DOI:** 10.3390/biomedicines13102477

**Published:** 2025-10-11

**Authors:** Huiying Wan, Ling Zhong, Jia Su, Qiaofeng Zhao, Mitsutoshi Tominaga, Kenji Takamori, Hang Ma, Tian Xia, Dingding Zhang

**Affiliations:** 1 Department of Dermatology, Sichuan Academy of Medical Sciences, Sichuan Provincial People’s Hospital, University of Electronic Science and Technology of China, Chengdu 610072, China; phoenixwhy@163.com (H.W.); 13219381068@163.com (L.Z.); sujia2024@163.com (J.S.); 2 Juntendo Itch Research Center (JIRC), Institute for Environmental and Gender-Specific Medicine, Juntendo University Graduate School of Medicine, Chiba 279-0021, Japan; tominaga@juntendo.ac.jp (M.T.); ktakamor@juntendo.ac.jp (K.T.); 3 Department of Biomedical and Pharmaceutical Sciences, College of Pharmacy, University of Rhode Island, Kingston, RI 02881, USA; hang_ma@uri.edu; 4 Department of Pathology, Air Force Hospital of Western Theater Command, Chengdu 610065, China; yours_summer@vip.sina.com; 5 Sichuan Provincial Key Laboratory for Genetic Disease, Sichuan Academy of Medical Sciences, Sichuan Provincial People’s Hospital, University of Electronic Science and Technology of China, Chengdu 610072, China

**Keywords:** medication use, cutaneous melanomas, mendelian randomization, genetics, single nucleotide polymorphisms

## Abstract

**Background/Objective:** Cutaneous melanoma is a highly heterogeneous malignancy and life-threatening skin cancer with rising global incidence. Although various therapeutic options are available, their clinical efficacy remains limited, highlighting the urgent need for novel strategies that facilitate prevention, diagnosis, and treatment. The aim of this study was to explore the potential causal association between medication use and the risk of developing cutaneous melanomas. **Methods:** Using summary data from Genome-Wide Association Studies (GWASs), we performed Mendelian randomization (MR) to investigate the causal effect of medication use on cutaneous melanoma risk. Exposure data were based on self-reported medication uses from ~320,000 European participants in the UK Biobank. The outcomes included GWAS results from 2824 cutaneous melanoma cases. Single-nucleotide polymorphisms (SNPs) significantly associated with medication use were used as instruments and analyzed with IVW, weighted median, weighted mode, and MR-Egger methods. Sensitivity analyses were used to assess pleiotropy and heterogeneity. **Results:** The analysis revealed that genetically predicted high use of adrenergics, inhalers, glucocorticoids, and opioids was suggestively associated with a reduced risk of cutaneous melanoma. Sensitivity analyses supported the robustness of these findings, showing no evidence of horizontal pleiotropy or influence from outliers. **Conclusions:** The results presented herein suggest that certain medication uses may lower the risk of developing cutaneous melanomas, offering potential new avenues for future prevention and treatment strategies.

## 1. Introduction

Cutaneous melanoma, the most aggressive type of skin cancer, arises from melanocytes and has seen a steady increase in incidence over recent decades. At a global scale, approximately 325,000 new cases of melanoma are reported annually [1]. Although melanoma constitutes only roughly 4% of all skin cancers, it accounts for 75% of skin cancer-related deaths [2]. Based on a 2019 Global Burden of Disease assessment, cutaneous melanoma ranked 16th out of 38 cancers in terms of disability-adjusted life years (DALYs) in the United States [3]. Despite advances in treatment [4], the disease burden imposed by melanoma remains substantial, underscoring the need to identify novel biomarkers to improve diagnosis, treatment, and prognosis.

The development of cutaneous melanoma is influenced by a complex interplay of genetic and environmental factors [5]. Among potential risk modifiers, medication use has emerged as an area of growing concern. Clinical observations and the results of epidemiological studies have suggested that some medications may alter melanoma risk; however, findings across different substances have been inconsistent. For instance, while the results of studies on β-blockers have suggested reduced metastasis, recurrence, and improved survival rates among cancer patients [6], the authors of other studies have not reported significant differences [7]. These inconsistencies highlight the need for in-depth investigations, particularly into medication effects specific to cutaneous melanoma.

Several medication classes have been examined to determine their impact on melanoma risk. Glucocorticoids, due to their anti-inflammatory effects, may theoretically reduce pro-carcinogenic inflammation and have been shown to inhibit melanoma cell proliferation at high doses [8]. Yet, long-term oral glucocorticoid use may slightly increase the risk of melanoma [9], and definitive evidence remains lacking. Opioids, commonly used for chronic pain, have been linked to increased risk of various cancers [10]. Although the results of some studies suggest that they may inhibit tumor angiogenesis through the suppression of VEGF signaling [11], their net effect on melanoma remains unclear. Non-steroidal anti-inflammatory drugs (NSAIDs) have demonstrated protective effects in some large cohort studies, with regular use associated with decreased skin cancer risk, including melanoma [12], possibly due to long-term suppression of tumor growth [13]. Statins, widely prescribed to treat hypercholesterolemia, may modulate inflammation and immune responses; however, their relationship with melanoma is controversial: the results of some cohort studies have demonstrated no significant effect [14], whereas the results of others have indicated increased risk with lipophilic statins and decreased risk of basal cell carcinoma with hydrophilic statins [15]. From the results presented above, while these medication classes have proven efficacy in other health contexts, their specific roles as risk or protective factors for cutaneous melanoma remain uncertain.

Given these conflicting findings, Mendelian randomization (MR) offers a powerful, unbiased approach to investigate potential causal relationships between medication use and melanoma risk. MR uses genetic variants as instrumental variables to proxy for exposures, enabling inference about causality while minimizing confounding and reverse causation [16]. MR has been increasingly applied in melanoma research to clarify the causal impact of various exposures on disease risk (see [17,18]). Recent MR studies have provided valuable insights into the genetic determinants of melanoma susceptibility and the causal roles of modifiable factors, emphasizing the value of this methodology in melanoma epidemiology.

In this context, we performed a two-sample MR analysis using publicly available GWAS datasets to systematically examine the potential causal associations between the use of 23 medication classes and the risk of cutaneous melanoma. We also conducted comprehensive sensitivity analyses to assess robustness. Through our findings, we aim to provide new perspectives on the prevention and management of cutaneous melanoma and to inform future research directions.

## 2. Materials and Methods

### 2.1. Research Framework

Our study follows the STrengthening the Reporting of Observational studies in Epidemiology using Mendelian Randomization (STROBE-MR) guidelines and is based on three core assumptions [19]:

(1) Relevance: The genetic instruments (IVs) are strongly associated with the exposure (medication use).
(2) Independence from confounders: The IVs are not associated with confounders of the exposure–outcome relationship.
(3) Exclusion restriction: The IVs affect the outcome (cutaneous melanoma) only through the exposure, not via alternative pathways.

Given the potential for confounding by indication in medication use traits, we further screened instruments for pleiotropy using LDtrait (see Section 2.6) and performed sensitivity analyses to evaluate the robustness of the exclusion restriction assumption.

No further ethical approval was required because the present study was based on publicly available GWAS data.

### 2.2. Data Acquisition

GWAS summary statistics for medication use were obtained from published sources [20] covering 23 medication categories in approximately 132,367 to 320,000 UK Biobank participants. To minimize sample overlap, the primary GWAS for cutaneous melanoma was sourced from FinnGen Release 12 (C3_MELANOMA_SKIN_EXALLC: 5753 cases and 378,749 controls), which does not include UK Biobank participants. Secondary analyses were performed using a UK Biobank-based melanoma GWAS (GCST90041829: 2824 cases and 453,524 controls) (Supplementary Table S1) [21]. Sample overlap between exposure and outcome GWASs was assessed and minimized; FinnGen results are reported as primary.

### 2.3. Instrumental Variable Selection

SNPs associated with the genome-wide significance of medication use and minor allele frequency (MAF) > 0.01 were screened. The threshold should satisfy *p* < 5 × 10^−8^ [22]. Based on R^2^ < 0.001 and a window size = 10,000 kb standard, we eliminate the linkage disequilibrium effect (LD) between SNPs [23]. When the selected IV does not exist in the summary data of the outcome, SNPS that have a high LD (R^2^ > 0.8) with this IV will be replaced as proxy SNPs. The *F*-value of each SNP in IV was calculated to evaluate the intensity of IV and to exclude the possible weak instrumental variable bias between IV and exposure factors. The calculation formula was as follows: *F* = R^2^ × (N − 2)/(1 − R^2^), where R^2^ is the proportion of variation in exposure that can be explained by the SNP in IV, and the requirement for the F-value is >10. Allele harmonization was performed to align effect alleles between the exposure and outcome datasets. Palindromic SNPs (A/T or C/G) with ambiguous strand orientation and an MAF close to 0.5 were excluded. Detailed harmonization procedures are described in the Supplementary Methods. For exposures with fewer than three valid IVs after harmonization and LD pruning, they were excluded from MR analysis to ensure the robustness of causal inference.

### 2.4. MR Analyses

We performed an MR analysis to investigate the causal association between medication use and cutaneous melanomas. Four commonly used MR methods are used for features containing multiple IVs: the random-effects inverse variance weighted method (IVW), weighted mode, weighted median estimation (WME), and MR-Egger regression. IVW is the most important and weighted mode method to interpret the results of MR; it takes the inverse variance of each SNP as the weight to calculate the weighted average of the effect size [24]. Therefore, IVW was used as the main analysis method to evaluate the causal association between medication use exposure and the risk of cutaneous melanoma outcomes by calculating the odds ratio (OR) and 95% confidence interval (CI). In addition, the MR-Egger, WME, and weighted mode methods were used to test the robustness of the analysis results [25]. The MR-Egger method can provide an accurate estimation of the causal effect in the case of pleiotropic bias because it considers the existence of an intercept term. The WME method assumes the validity of half of the IV to analyze the causal association between exposure and outcome. IVW was considered the primary method; results from the MR-Egger, weighted median, and weighted mode were used for sensitivity assessment. Results with FDR-adjusted *p* < 0.05 were considered statistically significant; *p* < 0.05 without FDR correction was considered suggestive evidence only. Where applicable, MR-RAPS (Robust Adjusted Profile Score) and Radial MR were also used to assess sensitivity to outlying instruments and horizontal pleiotropy. Given the multiple comparisons across 23 medications, we controlled the false discovery rate (FDR) using the Benjamini–Hochberg procedure. Associations were considered statistically significant at FDR < 0.05. Nominally significant results (*p* < 0.05, FDR ≥ 0.05) were described as “suggestive associations”.

### 2.5. Sensitivity Analysis

Sensitivity analysis is used to detect potential pleiotropy that may be present in MR studies. In this analysis, Cochran’s Q test, MR-Egger regression, MR-PRESSO, and leave-one-out methods were used to evaluate potential pleiotropy. Heterogeneity among IVs was assessed using Cochran’s Q test [26], and *p* > 0.05 was considered to be low heterogeneity, indicating that the estimates between IVs vary randomly, and the effect on IVW results is not significant. Secondly, considering that the pleiotropy of genetic variation may have an impact on the estimation of an association effect, we adopted the MR-Egger regression method to investigate the presence or absence of horizontal pleiotropy and determine the existence of pleiotropy by the intercept term of MR-Egger regression (when it approaches 0 or has no statistical significance, indicating that there is no pleiotropy) [27]. In addition, we also used the MR-PRESSO method to detect the possible presence of outliers (SNPs with *p* < 0.05) [28]. Radial MR was used for outlier and influential point detection, and MR-RAPS was used for robust effect estimates in the presence of weak instruments or outliers. Outliers detected using these methods were iteratively removed until all of the MR-PRESSO global test *p* > 0.05, MR-Egger Q-test *p* > 0.05, and MR-Egger intercept *p* > 0.05 were satisfied.

### 2.6. Instrument Pleiotropy and Confounder Screening

For exposures with nominally significant MR results, all instrument SNPs (and proxies) were cross-referenced in LDtrait (window ± 500 kb, R^2^ = 1) to identify associations with potential confounders, particularly pigmentation, sun exposure, and immune-related traits.

### 2.7. Additional Analyses and Statistical Corrections

To assess the validity of the causal direction from medication use to cutaneous melanoma, we performed the Steiger directionality test, which compares the variance explained in exposure and outcome by the selected instruments. We estimated the statistical power of our MR analyses using the mRnd online calculator (https://shiny.cnsgenomics.com/mRnd/, (accessed on 13 September 2025)). Minimum detectable odds ratios for various instrument strengths (R^2^) are presented in Supplementary Table S2 to contextualize the findings. All analyses were performed using R version 4.3.3. Forest plots, scatter plots, and funnel plots were produced with the TwoSampleMR and ggplot2 packages.

## 3. Results

### 3.1. Selection of Instrumental Variables (IVs)

**Figure 1:**
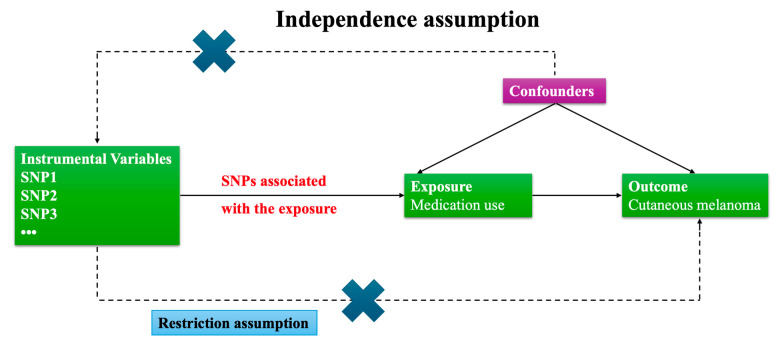
Flow chart of the study design. This flow chart outlines the overall study design and analytical workflow of the Mendelian randomization analysis. The diagram summarizes the selection of medication use exposures and melanoma outcome GWAS datasets, the identification and quality control of instrumental variables (IVs), data harmonization, main MR analysis, sensitivity analyses, and interpretation of the results.

A total of 910 independent SNPs were selected as instrumental variables (IVs) across 23 medication use categories (Figure 1 for flow chart of the study design; Supplementary Table S1 for exposure GWAS details). The mean F-statistics for all exposures ranged from 33.3 to 229.7, exceeding the conventional threshold of 10, indicating adequate instrument strength (Supplementary Table S2). Palindromic SNPs with ambiguous strand orientation were excluded during harmonization, and proxies (R^2^ > 0.8) were used where necessary, as described in the Methods section. The full list of IVs, F-statistic distributions, and details of removed outlier SNPs are provided in Supplementary Table S3.

Given the relatively modest number of melanoma cases in the GWAS datasets (FinnGen: 5753 cases; UK Biobank: 2824 cases), and the generally low variance explained (R^2^) for medication use by the genetic instruments, statistical power to detect modest effect sizes was limited for most exposures. For example, for agents acting on the renin–angiotensin system (R^2^ ≈ 0.07), statistical power for an OR of approximately 1.09 did not exceed 20% (Supplementary Table S2 for minimum detectable ORs and power for each exposure–outcome pair). To improve robustness, primary analyses were conducted using FinnGen melanoma GWAS data, with additional results for UK Biobank cutaneous melanoma. Exposures with fewer than 5 SNPs as IVs (e.g., opioids) are interpreted as exploratory.

### 3.2. Mendelian Randomization Analysis

**Figure 2:**
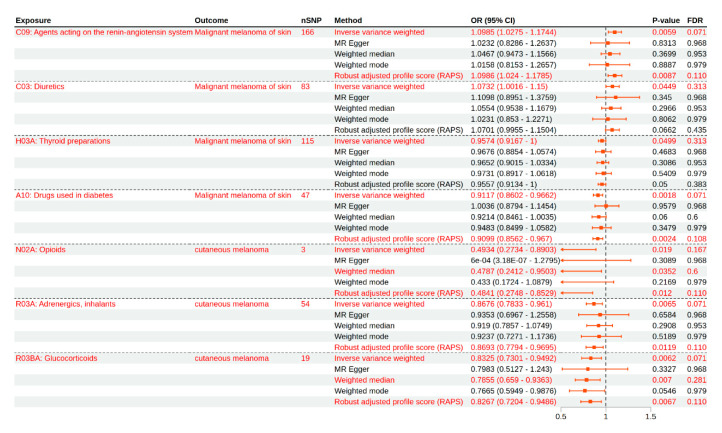
Mendelian randomization estimates for the associations between medication use and melanoma risk. This forest plot presents the odds ratios (ORs) and 95% confidence intervals (CIs) for the associations between genetically predicted medication use (by class) and melanoma outcomes, as estimated using different MR methods. The results are shown for the primary inverse variance weighted (IVW) analysis, in addition to the MR-Egger, weighted median, weighted mode, and robust adjusted profile score (RAPS) methods. Associations reaching suggestive significance after FDR correction are highlighted. Data are shown for both FinnGen (malignant melanoma of the skin) and UK Biobank (cutaneous melanoma) outcomes.

The MR analysis revealed several suggestive associations between genetically predicted medication use and melanoma outcomes (Table 1, Figure 2). In the FinnGen dataset, genetic liability to the use of agents acting on the renin–angiotensin system (C09) was suggestively associated with an increased risk of malignant melanoma. The IVW method estimated an OR of 1.10 (95% CI: 1.03–1.17, *p* = 0.0059, FDR = 0.0712, Supplementary Figure S1A–E), and this finding was consistent with the robust adjusted profile score (RAPS) method (OR = 1.10, 95% CI: 1.02–1.18, *p* = 0.0087). Diuretics (C03) also showed a suggestive association with higher malignant melanoma risk (IVW OR = 1.07, 95% CI: 1.00–1.15, *p* = 0.0449, FDR = 0.3138, Supplementary Figure S2A–E), though this did not reach the threshold for FDR significance. In contrast, thyroid preparations (H03A) were suggestively associated with a decreased risk of malignant melanoma (IVW OR = 0.96, 95% CI: 0.92–1.00, *p* = 0.0499, FDR = 0.3138), with other MR methods yielding consistent but non-significant results. Notably, the use of diabetes drugs (A10) was associated with a reduced risk of malignant melanoma (IVW OR = 0.91, 95% CI: 0.86–0.97, *p* = 0.0018, FDR = 0.0712, Supplementary Figure S3A–E), and this finding was supported by the RAPS method (OR = 0.91, 95% CI: 0.86–0.97, *p* = 0.0024).

For cutaneous melanoma outcomes in the UK Biobank dataset, several exposures demonstrated suggestive inverse associations. Genetically predicted higher use of adrenergics, inhalants (R03A), was associated with a lower risk of cutaneous melanoma (IVW OR = 0.87, 95% CI: 0.78–0.96, *p* = 0.0065, FDR = 0.0712, Supplementary Figure S4A–D), and this finding was corroborated by the RAPS method (OR = 0.84, 95% CI: 0.72–0.99, *p* = 0.0384). Similarly, genetically predicted glucocorticoid use (R03BA) was suggestively associated with a decreased risk of cutaneous melanoma (IVW OR = 0.86, 95% CI: 0.73–0.95, *p* = 0.0062, FDR = 0.0712, Supplementary Figure S5A–D). This association remained in the weighted median (OR = 0.77, 95% CI: 0.60–0.98, *p* = 0.0454) and RAPS (OR = 0.83, 95% CI: 0.72–0.95, *p* = 0.0067) analyses. For opioid use (N02A), although only three SNPs were available as instruments, higher genetically predicted exposure was suggestively associated with a reduced risk of cutaneous melanoma (IVW OR = 0.49, 95% CI: 0.27–0.89, *p* = 0.019, FDR = 0.167, Supplementary Figure S6A–D), with similar results from the weighted median (OR = 0.48, 95% CI: 0.26–0.89, *p* = 0.0206). However, given the limited number of IVs, this finding should be interpreted as exploratory.

Overall, after correction for multiple testing, the most consistent suggestive evidence was observed for increased risk of malignant melanoma with genetic liability to renin–angiotensin system agents and decreased risk of cutaneous melanoma with genetic liability to adrenergics, inhalants, and glucocorticoids. The results obtained using the different MR methods were generally consistent in direction, and all of the main findings are detailed in Table 1 and visualized in Figure 2. For exposures with fewer SNPs, such as opioids, the results require cautious interpretation due to limited instrument strength.

### 3.3. Sensitivity and Pleiotropy Analysis

Sensitivity analyses were conducted to assess the heterogeneity and horizontal pleiotropy across all primary associations (Table 1, Table 2 and Table 3). Heterogeneity was evaluated using Cochran’s Q statistic, whereas horizontal pleiotropy was assessed by the MR-Egger intercept. After outlier removal, no significant heterogeneity (Cochran’s Q *p* > 0.05) or horizontal pleiotropy (MR-Egger intercept *p* > 0.05) was detected for any main association (Table 2 and Table 3). For exposures with fewer than five SNPs, such as opioids, these tests have limited power, and the results should be interpreted with caution.

The robustness of the main positive findings was further supported by a series of sensitivity plots, including scatter, forest, funnel, and leave-one-out analyses, as presented in Supplementary Figures S1–S6. The Steiger directionality test further confirmed that the inferred causal direction was from medication use to melanoma risk for all exposure–outcome pairs (all *p* < 1 × 10^−5^; Table 4). In addition, MR-RAPS and Radial MR analyses demonstrated effect estimates that were consistent in direction and magnitude with those from the IVW and weighted median/mode methods (Table 3). For exposures with significant outliers, the effect sizes remained stable after outlier removal, supporting the robustness of the primary results.

## 4. Discussion

In this study, we used MR to explore the potential causal associations between genetic liability to specific medication use and the risk of cutaneous melanoma. Our findings provide suggestive evidence that genetic liability to the use of certain medications—particularly agents acting on the renin–angiotensin system, diuretics, thyroid preparations, and diabetes drugs in the FinnGen dataset and adrenergics, inhalants, glucocorticoids, and opioids in the UK Biobank dataset—may be associated with melanoma risk. However, these associations should be interpreted as exploratory and hypothesis-generating, rather than definitive evidence of causality, due to various limitations, including genetic instrument strength, potential confounding, and the modest effect sizes observed.

By leveraging both FinnGen and UK Biobank GWAS datasets, we sought to strengthen the robustness and generalizability of our results. Notably, the FinnGen-based associations involved medications typically prescribed for cardiovascular or metabolic conditions (e.g., agents acting on the renin–angiotensin system, diuretics, thyroid preparations, and diabetes drugs); in comparison, the UK Biobank-based associations were observed for medications more commonly used for pain or respiratory diseases (e.g., opioids, adrenergics, inhalants, and glucocorticoids). This lack of overlap between significant associations in the two datasets may reflect differences in phenotype definitions, comorbidity patterns, population genetic background, and environmental exposures, in addition to limitations in statistical power.

It is crucial to emphasize the relevance and strength of the genetic instruments used in MR. In our analysis, the average F-statistics for all exposures exceeded 10, suggesting adequate instrument strength and reducing the risk of weak instrument bias. However, for some exposures—particularly those with fewer than five SNPs (e.g., opioids)—the power to detect and correct for pleiotropy was limited, and such findings must be considered exploratory. Our power calculations, based on mRnd, highlight the limited ability to detect small effect sizes given the available sample sizes and instrument strength.

Our FinnGen-based results suggest that genetic liability to the use of agents acting on the renin–angiotensin system, diuretics, thyroid preparations, or diabetes drugs may be associated with melanoma risk. These medications are commonly prescribed for cardiovascular and metabolic diseases—conditions that themselves may be linked with skin cancer risk through shared risk factors (e.g., obesity, chronic inflammation, and metabolic dysregulation). The observed associations could be influenced by underlying disease liability or by pleiotropic genetic effects and thus require cautious interpretation and further validation.

For adrenergics, inhalants, and glucocorticoids—primarily used in asthma—our findings are consistent with some preclinical studies suggesting potential biological pathways linking these medications and melanoma biology [29,30,31,32,33,34]. For example, β2-adrenergic receptor agonists can modulate intracellular cAMP in melanoma cells [31], and glucocorticoids may affect cell survival and immune interactions [8,35]. However, these findings from cell lines or animal models should not be assumed to translate directly to human population effects; cultured cells behave differently than cells in vivo, and results at the population level are subject to complex confounding.

With opioids, the observed association (in the UK Biobank dataset) was based on only three SNPs and should be regarded as exploratory. While the results of previous studies have suggested that opioid use may be linked to cancer risk through immune modulation or angiogenesis inhibition [10,11,36], its clinical relevance for melanoma is uncertain; thus, further research is needed.

It is well established that the vast majority of melanomas occur in individuals with fair skin (low melanin), high nevus counts, or a genetic predisposition, particularly those with substantial ultraviolet (UV) exposure or severe sunburns [37]. Environmental and inherited factors remain the dominant determinants of melanoma risk [38]. The potential impact of medication use on melanoma risk is likely to be much smaller than these major risk factors [39]. With our findings, we aim to uncover new, potentially modifiable or targetable biological pathways beyond the primary environmental and genetic determinants, rather than to suggest that medication use is a dominant factor.

Interpretation of these associations must be cautious, as medication use often reflects underlying disease states (e.g., asthma, cardiovascular disease, diabetes, and chronic pain), which themselves may influence melanoma risk through shared mechanisms or confounding [40]. Rates of diseases such as respiratory disorders, cardiovascular disease, diabetes, obesity, and orthopedic or autoimmune conditions can affect both the likelihood of medication exposure and the baseline melanoma risk [41]. Social habits (e.g., outdoor activity, sun protection, and occupational exposures) and unmeasured environmental factors may also play crucial roles. Our MR approach partially addresses confounding but cannot fully disentangle these complex relationships, particularly with polygenic traits and pleiotropic loci.

It is critical to underscore that MR estimates reflect the effect of genetic liability to medication use, not the direct pharmacologic effect of actual drug exposure. The findings of this study must not be interpreted as a recommendation to use these medications for melanoma prevention. Both glucocorticoids and opioids have well-documented and potentially serious side effects, including immunosuppression, metabolic disturbances, addiction, and increased risk of infection or other cancers [42]. The goal of this research is to identify biological pathways that may be targeted in the future by safer interventions, not to advocate for increased use of these specific drugs.

Our study population consisted of individuals of broadly European ancestry. However, there is substantial heterogeneity within European populations regarding skin pigmentation, nevus density, sun sensitivity, environmental exposures, and social habits. Individuals of Northern European descent (e.g., English, Irish, Scottish, and Scandinavian) with lighter skin are at the highest melanoma risk due to both genetic and environmental factors [41]. In our analysis, we could not stratify by these finer subgroups, and the findings may not generalize to non-European or admixed populations. More diverse studies are needed to understand effect modification by ancestry, skin type, and environment.

The two-sample MR design used in this study leveraged data from large-scale GWASs, effectively excluding confounding factors and providing relatively unbiased causal evidence [16]. However, the study has its limitations. First, the number of IVs was limited for certain medication exposures (e.g., opioids, with only three SNPs), resulting in reduced statistical power and restricting the ability to robustly assess heterogeneity and horizontal pleiotropy using sensitivity analyses. For all exposures with fewer than five IVs, the findings should be interpreted as exploratory rather than confirmatory. Second, while we applied multiple robust MR methods (including random-effects IVW, MR-Egger, and MR-PRESSO), the accuracy of pleiotropy detection and correction is inherently limited when the number of instruments is low. Third, our analyses rely solely on bioinformatic approaches using publicly available GWAS summary statistics and are not accompanied by experimental or mechanistic validation. Future studies integrating laboratory-based functional assays or clinical data are needed to support and clarify the biological mechanisms underlying these genetic associations. Fourth, medication use was primarily self-reported in the original GWAS, which may introduce misclassification or recall bias and reduce the reliability of exposure data.

Fifth, although the GWAS data were described as being from individuals of European ancestry, there is considerable heterogeneity within European populations in terms of skin pigmentation, environmental exposures (e.g., UV radiation), and social habits, all of which may influence melanoma risk. In our study, we were unable to account for these internal population differences, and the findings may not generalize to non-European or more diverse populations. Lastly, while the random-effects IVW model can accommodate some degree of heterogeneity, results for exposures or outcomes with significant heterogeneity should still be interpreted with caution.

The authors of future studies should aim to validate these findings in larger, more diverse cohorts and ideally incorporate prospective or experimental approaches to provide mechanistic insights. Stratified analyses by finer ancestry groups, skin type, and environmental factors would improve our understanding of gene–environment interactions. Ultimately, a deeper understanding of these associations could help develop personalized medicine strategies to reduce melanoma risk without compromising treatment efficacy.

## 5. Conclusions

In conclusion, through this study, we provide new evidence of a potential causal association between medication use and the risk of cutaneous melanoma, particularly with adrenergics, inhalants, glucocorticoids, and opioids. These findings not only offer new biological insights into the association between medication use and cutaneous melanoma but also provide possible targets for developing new prevention and treatment strategies. The authors of future studies should explore in greater depth the mechanisms of action of these medications and validate their effectiveness and safety in clinical practice. Such efforts are critical for guiding clinical recommendations and improving patient outcomes.

## Figures and Tables

**Table 1:** Mendelian randomization results for all exposures and melanoma outcomes.

Exposure	Outcome	Number of SNPs	Method	OR (95% CI)	*p*-Value	FDR
C09: Agents acting on the renin–angiotensin system	Malignant melanoma of the skin	166	Inverse variance weighted	1.0985 (1.0275–1.1744)	0.0059	0.0712
			MR Egger	1.0232 (0.8286–1.2637)	0.8313	0.9686
			Weighted median	1.0467 (0.9473–1.1566)	0.3699	0.9535
			Weighted mode	1.0158 (0.8153–1.2657)	0.8887	0.9795
			Robust adjusted profile score (RAPS)	1.0986 (1.024–1.1785)	0.0087	0.1108
C09: Agents acting on the renin–angiotensin system	Cutaneous melanoma	165	Inverse variance weighted	1.0385 (0.9452–1.141)	0.4316	0.8291
			MR Egger	0.82 (0.6154–1.0927)	0.1774	0.9089
			Weighted median	1.008 (0.8769–1.1586)	0.9108	0.9535
			Weighted mode	0.9473 (0.6385–1.4054)	0.7882	0.9795
			Robust adjusted profile score (RAPS)	1.0416 (0.9438–1.1495)	0.4181	0.9159
C03: Diuretics	Malignant melanoma of the skin	83	Inverse variance weighted	1.0732 (1.0016–1.15)	0.0449	0.3138
			MR Egger	1.1098 (0.8951–1.3759)	0.345	0.9686
			Weighted median	1.0554 (0.9538–1.1679)	0.2966	0.9535
			Weighted mode	1.0231 (0.853–1.2271)	0.8062	0.9795
			Robust adjusted profile score (RAPS)	1.0701 (0.9955–1.1504)	0.0662	0.4351
C03: Diuretics	Cutaneous melanoma	89	Inverse variance weighted	1.0318 (0.9321–1.1421)	0.5463	0.9244
			MR Egger	1.0956 (0.7872–1.5249)	0.5896	0.9686
			Weighted median	0.9406 (0.8081–1.0949)	0.4296	0.9535
			Weighted mode	0.7402 (0.5086–1.0771)	0.1195	0.9795
			Robust adjusted profile score (RAPS)	1.0321 (0.9201–1.1577)	0.59	0.9642
C08: Calcium channel blockers	Malignant melanoma of the skin	89	Inverse variance weighted	1.0619 (0.992–1.1367)	0.0837	0.3763
			MR Egger	1.1229 (0.9061–1.3917)	0.2925	0.9686
			Weighted median	1.0317 (0.9307–1.1436)	0.5529	0.9535
			Weighted mode	1.0551 (0.8944–1.2447)	0.5264	0.9795
			Robust adjusted profile score (RAPS)	1.0569 (0.9841–1.135)	0.1285	0.4748
C08: Calcium channel blockers	Cutaneous melanoma	92	Inverse variance weighted	0.9871 (0.896–1.0875)	0.7928	0.9357
			MR Egger	0.9797 (0.722–1.3293)	0.8954	0.9686
			Weighted median	0.9929 (0.8585–1.1483)	0.9232	0.9535
			Weighted mode	1.0083 (0.7256–1.4013)	0.9607	0.9795
			Robust adjusted profile score (RAPS)	0.9824 (0.8874–1.0875)	0.7316	0.9642
H03A: Thyroid preparations	Malignant melanoma of the skin	115	Inverse variance weighted	0.9574 (0.9167–1)	0.0499	0.3138
			MR Egger	0.9676 (0.8854–1.0574)	0.4683	0.9686
			Weighted median	0.9652 (0.9015–1.0334)	0.3086	0.9535
			Weighted mode	0.9731 (0.8917–1.0618)	0.5409	0.9795
			Robust adjusted profile score (RAPS)	0.9557 (0.9134–1)	0.05	0.383
H03A: Thyroid preparations	Cutaneous melanoma	116	Inverse variance weighted	0.9477 (0.89–1.0092)	0.0941	0.3763
			MR Egger	0.9105 (0.7885–1.0514)	0.2041	0.9089
			Weighted median	0.9146 (0.8248–1.014)	0.09	0.7203
			Weighted mode	0.8998 (0.7953–1.0179)	0.0962	0.9795
			Robust adjusted profile score (RAPS)	0.9436 (0.8838–1.0075)	0.0825	0.4461
C07: Beta blocking agents	Malignant melanoma of the skin	57	Inverse variance weighted	1.0629 (0.9584–1.1787)	0.2478	0.6413
			MR Egger	1.3246 (0.8948–1.9609)	0.1658	0.9089
			Weighted median	1.0111 (0.889–1.15)	0.8665	0.9535
			Weighted mode	0.9685 (0.7773–1.2068)	0.7765	0.9795
			Robust adjusted profile score (RAPS)	1.0586 (0.9553–1.173)	0.2769	0.7077
C07: Beta blocking agents	Cutaneous melanoma	54	Inverse variance weighted	1.0116 (0.8876–1.153)	0.8625	0.9357
			MR Egger	0.9781 (0.6107–1.5665)	0.927	0.9686
			Weighted median	0.9744 (0.8045–1.1803)	0.7911	0.9535
			Weighted mode	0.8886 (0.5475–1.4423)	0.6346	0.9795
			Robust adjusted profile score (RAPS)	1.024 (0.8923–1.1752)	0.7356	0.9642
C10AA: HMG CoA reductase inhibitors	Malignant melanoma of the skin	87	Inverse variance weighted	1.0653 (0.9842–1.1531)	0.1175	0.3977
			MR Egger	0.997 (0.8595–1.1566)	0.9686	0.9686
			Weighted median	1.0795 (0.9548–1.2205)	0.2217	0.9535
			Weighted mode	1.0715 (0.9276–1.2378)	0.3505	0.9795
			Robust adjusted profile score (RAPS)	1.0585 (0.9747–1.1495)	0.1767	0.4782
C10AA: HMG CoA reductase inhibitors	Cutaneous melanoma	85	Inverse variance weighted	1.0866 (0.9751–1.2107)	0.1326	0.4167
			MR Egger	1.0268 (0.8457–1.2467)	0.7899	0.9686
			Weighted median	1.0098 (0.8455–1.206)	0.914	0.9535
			Weighted mode	1.0504 (0.8504–1.2973)	0.6496	0.9795
			Robust adjusted profile score (RAPS)	1.0962 (0.9796–1.2267)	0.1093	0.4572
A10: Drugs used in diabetes	Malignant melanoma of the skin	47	Inverse variance weighted	0.9117 (0.8602–0.9662)	0.0018	0.0712
			MR Egger	1.0036 (0.8794–1.1454)	0.9579	0.9686
			Weighted median	0.9214 (0.8461–1.0035)	0.06	0.6
			Weighted mode	0.9483 (0.8499–1.0582)	0.3479	0.9795
			Robust adjusted profile score (RAPS)	0.9099 (0.8562–0.967)	0.0024	0.1083
A10: Drugs used in diabetes	Cutaneous melanoma	49	Inverse variance weighted	0.9883 (0.9134–1.0693)	0.7698	0.9357
			MR Egger	0.9475 (0.7921–1.1334)	0.5578	0.9686
			Weighted median	0.9964 (0.8829–1.1245)	0.9535	0.9535
			Weighted mode	0.994 (0.8677–1.1388)	0.9317	0.9795
			Robust adjusted profile score (RAPS)	0.9871 (0.9089–1.072)	0.7576	0.9642
N02A: Opioids	Malignant melanoma of the skin	3	Inverse variance weighted	0.849 (0.5965–1.2086)	0.3636	0.8
			MR Egger	2.0287 (0.0016–2618.2156)	0.8783	0.9686
			Weighted median	0.9264 (0.5858–1.4651)	0.7436	0.9535
			Weighted mode	1.0082 (0.5805–1.7509)	0.9795	0.9795
			Robust adjusted profile score (RAPS)	0.847 (0.581–1.2349)	0.3881	0.8926
N02A: Opioids	Cutaneous melanoma	3	Inverse variance weighted	0.4934 (0.2734–0.8903)	0.019	0.167
			MR Egger	6 × 10^−4^ (3.18 × 10^−7^–1.2795)	0.3089	0.9686
			Weighted median	0.4787 (0.2412–0.9503)	0.0352	0.6
			Weighted mode	0.433 (0.1724–1.0879)	0.2169	0.9795
			Robust adjusted profile score (RAPS)	0.4841 (0.2748–0.8529)	0.012	0.1108
C02: Antihypertensives	Malignant melanoma of the skin	4	Inverse variance weighted	0.9702 (0.8149–1.155)	0.7337	0.9357
			MR Egger	0.341 (0.0019–60.0168)	0.7229	0.9686
			Weighted median	0.9523 (0.7857–1.1543)	0.6187	0.9535
			Weighted mode	0.9422 (0.7246–1.2252)	0.6871	0.9795
			Robust adjusted profile score (RAPS)	0.9701 (0.8044–1.1701)	0.7511	0.9642
C02: Antihypertensives	Cutaneous melanoma	4	Inverse variance weighted	0.8924 (0.6476–1.2296)	0.4863	0.8916
			MR Egger	0.038 (8.01 × 10^−7^–1799.4876)	0.6119	0.9686
			Weighted median	0.9098 (0.662–1.2502)	0.5599	0.9535
			Weighted mode	0.9436 (0.5583–1.5948)	0.8423	0.9795
			Robust adjusted profile score (RAPS)	0.8882 (0.6408–1.231)	0.4765	0.9642
C01D: Vasodilators used in cardiac diseases	Malignant melanoma of the skin	2	Inverse variance weighted	1.0158 (0.7987–1.2919)	0.8983	0.9357
			Robust adjusted profile score (RAPS)	1.0159 (0.7892–1.3077)	0.9024	0.9642
C01D: Vasodilators used in cardiac diseases	Cutaneous melanoma	2	Inverse variance weighted	0.8952 (0.5749–1.394)	0.6242	0.9357
			Robust adjusted profile score (RAPS)	0.967 (0.6625–1.4114)	0.8618	0.9642
M01A: Anti-inflammatory and antirheumatic products, non-steroids	Malignant melanoma of the skin	6	Inverse variance weighted	0.9754 (0.6532–1.4566)	0.9032	0.9357
			MR Egger	0.61 (0.0274–13.5924)	0.7705	0.9686
			Weighted median	0.9435 (0.5861–1.519)	0.811	0.9535
			Weighted mode	0.7593 (0.3974–1.4508)	0.4425	0.9795
			Robust adjusted profile score (RAPS)	0.968 (0.6284–1.4912)	0.8828	0.9642
M01A: Anti-inflammatory and antirheumatic products, non-steroids	Cutaneous melanoma	4	Inverse variance weighted	1.0399 (0.5307–2.038)	0.9091	0.9357
			MR Egger	0.8255 (0.0049–138.6755)	0.9482	0.9686
			Weighted median	1.1797 (0.5594–2.4878)	0.6643	0.9535
			Weighted mode	1.4986 (0.6099–3.6819)	0.4427	0.9795
			Robust adjusted profile score (RAPS)	1.0412 (0.5297–2.0468)	0.9068	0.9642
R03A: Adrenergics, inhalants	Malignant melanoma of the skin	48	Inverse variance weighted	0.9374 (0.871–1.0089)	0.0849	0.3763
			MR Egger	0.9678 (0.788–1.1887)	0.7565	0.9686
			Weighted median	0.9664 (0.8672–1.077)	0.5365	0.9535
			Weighted mode	0.9913 (0.847–1.1601)	0.9134	0.9795
			Robust adjusted profile score (RAPS)	0.9353 (0.8662–1.0098)	0.0873	0.4461
R03A: Adrenergics, inhalants	Cutaneous melanoma	54	Inverse variance weighted	0.8676 (0.7833–0.961)	0.0065	0.0712
			MR Egger	0.9353 (0.6967–1.2558)	0.6584	0.9686
			Weighted median	0.919 (0.7857–1.0749)	0.2908	0.9535
			Weighted mode	0.9237 (0.7271–1.1736)	0.5189	0.9795
			Robust adjusted profile score (RAPS)	0.8693 (0.7794–0.9695)	0.0119	0.1108
R03BA: Glucocorticoids	Malignant melanoma of the skin	17	Inverse variance weighted	0.9509 (0.8616–1.0494)	0.3169	0.734
			MR Egger	0.7243 (0.5201–1.0088)	0.0757	0.9089
			Weighted median	0.923 (0.804–1.0595)	0.2549	0.9535
			Weighted mode	0.8363 (0.6806–1.0276)	0.1083	0.9795
			Robust adjusted profile score (RAPS)	0.9444 (0.8478–1.0519)	0.2979	0.7213
R03BA: Glucocorticoids	Cutaneous melanoma	19	Inverse variance weighted	0.8325 (0.7301–0.9492)	0.0062	0.0712
			MR Egger	0.7983 (0.5127–1.243)	0.3327	0.9686
			Weighted median	0.7855 (0.659–0.9363)	0.007	0.2817
			Weighted mode	0.7665 (0.5949–0.9876)	0.0546	0.9795
			Robust adjusted profile score (RAPS)	0.8267 (0.7204–0.9486)	0.0067	0.1108
M05B: Drugs affecting bone structure and mineralization	Malignant melanoma of the skin	11	Inverse variance weighted	1.032 (0.8843–1.2045)	0.6893	0.9357
			MR Egger	0.6195 (0.3002–1.2781)	0.2272	0.9089
			Weighted median	1.0158 (0.8612–1.1983)	0.8521	0.9535
			Weighted mode	1.052 (0.8186–1.3519)	0.7005	0.9795
			Robust adjusted profile score (RAPS)	1.015 (0.8611–1.1964)	0.8588	0.9642
M05B: Drugs affecting bone structure and mineralization	Cutaneous melanoma	11	Inverse variance weighted	0.8885 (0.7549–1.0457)	0.1549	0.4259
			MR Egger	0.5807 (0.2663–1.2662)	0.2049	0.9089
			Weighted median	0.8601 (0.6909–1.0708)	0.1776	0.9535
			Weighted mode	0.8344 (0.5909–1.1782)	0.3279	0.9795
			Robust adjusted profile score (RAPS)	0.8868 (0.7457–1.0545)	0.1741	0.4782
S01E: Antiglaucoma preparations and miotics	Malignant melanoma of the skin	12	Inverse variance weighted	1.0028 (0.9331–1.0778)	0.9383	0.9383
			MR Egger	1.1416 (0.9417–1.3839)	0.2073	0.9089
			Weighted median	1.0056 (0.9113–1.1097)	0.9107	0.9535
			Weighted mode	1.0237 (0.9139–1.1467)	0.6936	0.9795
			Robust adjusted profile score (RAPS)	1.0021 (0.9254–1.0851)	0.9588	0.9642
S01E: Antiglaucoma preparations and miotics	Cutaneous melanoma	12	Inverse variance weighted	1.0186 (0.9121–1.1374)	0.744	0.9357
			MR Egger	0.75 (0.549–1.0246)	0.1008	0.9089
			Weighted median	0.9372 (0.8125–1.0811)	0.3733	0.9535
			Weighted mode	0.8963 (0.7495–1.0719)	0.2556	0.9795
			Robust adjusted profile score (RAPS)	1.016 (0.901–1.1457)	0.7958	0.9642
N02BA: Salicylic acid and derivatives	Malignant melanoma of the skin	7	Inverse variance weighted	0.9535 (0.721–1.2609)	0.7382	0.9357
			MR Egger	0.8693 (0.3767–2.0057)	0.7559	0.9686
			Weighted median	0.9498 (0.6512–1.3854)	0.7892	0.9535
			Weighted mode	0.9272 (0.5568–1.5439)	0.7811	0.9795
			Robust adjusted profile score (RAPS)	0.9404 (0.6985–1.266)	0.6855	0.9642
N02BA: Salicylic acid and derivatives	Cutaneous melanoma	10	Inverse variance weighted	0.9376 (0.6488–1.355)	0.7318	0.9357
			MR Egger	1.3303 (0.4619–3.8312)	0.6112	0.9686
			Weighted median	1.0405 (0.6674–1.6222)	0.8608	0.9535
			Weighted mode	1.13 (0.491–2.6004)	0.7804	0.9795
			Robust adjusted profile score (RAPS)	0.9448 (0.6355–1.4047)	0.7791	0.9642
R06A: Antihistamines for systemic use	Malignant melanoma of the skin	8	Inverse variance weighted	1.0232 (0.8421–1.2432)	0.8177	0.9357
			MR Egger	0.8562 (0.2704–2.7115)	0.8007	0.9686
			Weighted median	1.0115 (0.7952–1.2866)	0.926	0.9535
			Weighted mode	1.0153 (0.7312–1.4097)	0.9304	0.9795
			Robust adjusted profile score (RAPS)	1.0208 (0.8365–1.2457)	0.8395	0.9642
R06A: Antihistamines for systemic use	Cutaneous melanoma	8	Inverse variance weighted	0.9595 (0.7553–1.2191)	0.7352	0.9357
			MR Egger	2.9939 (0.8739–10.2575)	0.1315	0.9089
			Weighted median	0.9149 (0.6649–1.2588)	0.5848	0.9535
			Weighted mode	0.7714 (0.4687–1.2696)	0.3412	0.9795
			Robust adjusted profile score (RAPS)	0.9603 (0.744–1.2395)	0.756	0.9642
A02B: Drugs for peptic ulcers and gastro-oesophageal reflux disease (GORD)	Malignant melanoma of the skin	5	Inverse variance weighted	1.0987 (0.6533–1.8478)	0.7227	0.9357
			MR Egger	0.0676 (0.0032–1.4381)	0.1826	0.9089
			Weighted median	1.1083 (0.6519–1.884)	0.7042	0.9535
			Weighted mode	1.0863 (0.5387–2.1905)	0.8284	0.9795
			Robust adjusted profile score (RAPS)	1.0484 (0.6058–1.8143)	0.866	0.9642
A02B: Drugs for peptic ulcers and gastro-oesophageal reflux disease (GORD)	Cutaneous melanoma	5	Inverse variance weighted	0.6647 (0.4015–1.1005)	0.1124	0.3977
			MR Egger	0.5052 (0.0213–11.9822)	0.701	0.9686
			Weighted median	0.7571 (0.399–1.4366)	0.3945	0.9535
			Weighted mode	0.8053 (0.3286–1.9736)	0.6606	0.9795
			Robust adjusted profile score (RAPS)	0.6618 (0.3856–1.1359)	0.1342	0.4748
N02BE: Anilides	Malignant melanoma of the skin	6	Inverse variance weighted	0.8311 (0.536–1.2886)	0.4083	0.8291
			MR Egger	1.1435 (0.0541–24.1589)	0.9355	0.9686
			Weighted median	0.9822 (0.5975–1.6144)	0.9434	0.9535
			Weighted mode	0.9856 (0.4704–2.0653)	0.9709	0.9795
			Robust adjusted profile score (RAPS)	0.8657 (0.5328–1.4063)	0.5601	0.9642
N02BE: Anilides	Cutaneous melanoma	7	Inverse variance weighted	0.7463 (0.4402–1.265)	0.2771	0.6773
			MR Egger	2.6883 (0.0802–90.1552)	0.6048	0.9686
			Weighted median	0.5378 (0.2877–1.0053)	0.052	0.6
			Weighted mode	0.4773 (0.1795–1.2697)	0.189	0.9795
			Robust adjusted profile score (RAPS)	0.6967 (0.4133–1.1743)	0.1749	0.4782
N02C: Antimigraine preparations	Malignant melanoma of the skin	13	Inverse variance weighted	1.0387 (0.9446–1.1421)	0.4334	0.8291
			MR Egger	0.9788 (0.5059–1.894)	0.9505	0.9686
			Weighted median	1.0073 (0.8976–1.1304)	0.9015	0.9535
			Weighted mode	0.9935 (0.8373–1.1789)	0.9418	0.9795
			Robust adjusted profile score (RAPS)	1.0193 (0.9333–1.1132)	0.6709	0.9642
N02C: Antimigraine preparations	Cutaneous melanoma	13	Inverse variance weighted	1.0382 (0.928–1.1615)	0.5131	0.903
			MR Egger	0.7122 (0.3382–1.4998)	0.3908	0.9686
			Weighted median	1.0652 (0.9139–1.2414)	0.419	0.9535
			Weighted mode	1.0876 (0.8315–1.4227)	0.5513	0.9795
			Robust adjusted profile score (RAPS)	1.0369 (0.9212–1.1671)	0.5484	0.9642
N06A: Antidepressants	Malignant melanoma of the skin	1	Robust adjusted profile score (RAPS)	0.7943 (0.3369–1.8727)	0.5986	0.9642
N06A: Antidepressants	Cutaneous melanoma	1	Robust adjusted profile score (RAPS)	1.0533 (0.322–3.4458)	0.9315	0.9642
B01A: Antithrombotic agents	Malignant melanoma of the skin	9	Inverse variance weighted	1.0172 (0.789–1.3113)	0.8956	0.9357
			MR Egger	0.8049 (0.3888–1.6663)	0.5771	0.9686
			Weighted median	0.9784 (0.7018–1.3639)	0.8974	0.9535
			Weighted mode	0.9669 (0.563–1.6605)	0.9059	0.9795
			Robust adjusted profile score (RAPS)	1.0107 (0.775–1.3181)	0.9372	0.9642
B01A: Antithrombotic agents	Cutaneous melanoma	13	Inverse variance weighted	0.9836 (0.7278–1.3294)	0.9144	0.9357
			MR Egger	1.3796 (0.5997–3.1742)	0.4649	0.9686
			Weighted median	1.0373 (0.6939–1.5508)	0.8582	0.9535
			Weighted mode	1.3109 (0.5537–3.1038)	0.5496	0.9795
			Robust adjusted profile score (RAPS)	0.9928 (0.7232–1.3627)	0.9642	0.9642
L04: Immunosuppressants	Malignant melanoma of the skin	2	Inverse variance weighted	0.9325 (0.8605–1.0106)	0.0886	0.3763
			Robust adjusted profile score (RAPS)	0.9324 (0.8579–1.0134)	0.0996	0.4572
L04: Immunosuppressants	Cutaneous melanoma	2	Inverse variance weighted	0.9228 (0.8283–1.028)	0.1448	0.4247
			Robust adjusted profile score (RAPS)	0.9228 (0.8252–1.0319)	0.1585	0.4782

Odds ratios (ORs) and 95% confidence intervals (CIs) are shown for each method. FDR denotes the false discovery rate. Results based on a small number of SNPs should be interpreted as exploratory.

**Table 2:** MR-PRESSO results for main exposures and melanoma outcomes after outlier removal.

Exposure	Outcome	Raw		Global Test *p*-Value
		OR (95% CI)	*p*-Value	
C09: Agents acting on the renin–angiotensin system	Malignant melanoma of the skin	1.0985 (1.0302–1.1714)	0.0047	0.7398
C09: Agents acting on the renin–angiotensin system	Cutaneous melanoma	1.0385 (0.9497–1.1356)	0.4088	0.8078
C03: Diuretics	Malignant melanoma of the skin	1.0732 (1.0057–1.1454)	0.0361	0.7728
C03: Diuretics	Cutaneous melanoma	1.0318 (0.9321–1.1421)	0.5478	0.275
C08: Calcium channel blockers	Malignant melanoma of the skin	1.0619 (0.9951–1.1331)	0.0734	0.7192
C08: Calcium channel blockers	Cutaneous melanoma	0.9871 (0.9047–1.0770)	0.7710	0.9045
H03A: Thyroid preparations	Malignant melanoma of the skin	0.9574 (0.9234–0.9927)	0.0200	0.993
H03A: Thyroid preparations	Cutaneous melanoma	0.9477 (0.8954–1.0030)	0.0661	0.9348
C07: Beta blocking agents	Malignant melanoma of the skin	1.0629 (0.9584–1.1787)	0.2527	0.0752
C07: Beta blocking agents	Cutaneous melanoma	1.0116 (0.8981–1.1396)	0.8498	0.805
C10AA: HMG CoA reductase inhibitors	Malignant melanoma of the skin	1.0653 (0.9964–1.1390)	0.0670	0.9815
C10AA: HMG CoA reductase inhibitors	Cutaneous melanoma	1.0866 (0.9872–1.1959)	0.0934	0.9247
A10: Drugs used in diabetes	Malignant melanoma of the skin	0.9117 (0.8672–0.9585)	0.0007	0.9018
A10: Drugs used in diabetes	Cutaneous melanoma	0.9883 (0.9158–1.0665)	0.7635	0.6068
C02: Antihypertensives	Malignant melanoma of the skin	0.9702 (0.9249–1.0177)	0.3029	0.975
C02: Antihypertensives	Cutaneous melanoma	0.8924 (0.6476–1.2296)	0.5364	0.2502
M01A: Anti-inflammatory and antirheumatic products, non-steroids	Malignant melanoma of the skin	0.9754 (0.6532–1.4566)	0.9079	0.3383
M01A: Anti-inflammatory and antirheumatic products, non-steroids	Cutaneous melanoma	1.0399 (0.5307–2.0380)	0.9164	0.3513
R03A: Adrenergics, inhalants	Malignant melanoma of the skin	0.9374 (0.8726–1.0070)	0.0834	0.5812
R03A: Adrenergics, inhalants	Cutaneous melanoma	0.8676 (0.7833–0.9610)	0.0087	0.4147
R03BA: Glucocorticoids	Malignant melanoma of the skin	0.9509 (0.8623–1.0487)	0.3283	0.4442
R03BA: Glucocorticoids	Cutaneous melanoma	0.8325 (0.7560–0.9167)	0.0015	0.9403
M05B: Drugs affecting bone structure and mineralization	Malignant melanoma of the skin	1.0320 (0.8843–1.2045)	0.6977	0.1475
M05B: Drugs affecting bone structure and mineralization	Cutaneous melanoma	0.8885 (0.7788–1.0136)	0.1090	0.7827
S01E: Antiglaucoma preparations and miotics	Malignant melanoma of the skin	1.0028 (0.9331–1.0778)	0.9397	0.3787
S01E: Antiglaucoma preparations and miotics	Cutaneous melanoma	1.0186 (0.9121–1.1374)	0.7501	0.288
N02BA: Salicylic acid and derivatives	Malignant melanoma of the skin	0.9535 (0.7210–1.2609)	0.7495	0.4637
N02BA: Salicylic acid and derivatives	Cutaneous melanoma	0.9376 (0.6488–1.3550)	0.7397	0.1655
R06A: Antihistamines for systemic use	Malignant melanoma of the skin	1.0232 (0.8421–1.2432)	0.8243	0.312
R06A: Antihistamines for systemic use	Cutaneous melanoma	0.9595 (0.7597–1.2120)	0.7391	0.495
A02B: Drugs for peptic ulcers and gastro-oesophageal reflux disease (GORD)	Malignant melanoma of the skin	1.0987 (0.6533–1.8478)	0.7406	0.151
A02B: Drugs for peptic ulcers and gastro-oesophageal reflux disease (GORD)	Cutaneous melanoma	0.6647 (0.4653–0.9497)	0.0883	0.7485
N02BE: Anilides	Malignant melanoma of the skin	0.8311 (0.5360–1.2886)	0.4459	0.2148
N02BE: Anilides	Cutaneous melanoma	0.7463 (0.4402–1.2650)	0.3188	0.2253
N02C: Antimigraine preparations	Malignant melanoma of the skin	1.0387 (0.9446–1.1421)	0.4486	0.1557
N02C: Antimigraine preparations	Cutaneous melanoma	1.0382 (0.9461–1.1391)	0.4444	0.7472
B01A: Antithrombotic agents	Malignant melanoma of the skin	1.0172 (0.8252–1.2537)	0.8773	0.6837
B01A: Antithrombotic agents	Cutaneous melanoma	0.9836 (0.7278–1.3294)	0.9162	0.3003

Heterogeneity was assessed using Cochran’s Q statistic. Horizontal pleiotropy was assessed using the MR-Egger intercept.

**Table 3:** Sensitivity analyses after outlier removal.

Exposure	Outcome	Heterogeneity		Pleotropy	
		Q Statistic (IVW)	*p*-Value	MR-Egger Intercept	*p*-Value
C09: Agents acting on the renin–angiotensin system	Malignant melanoma of the skin	152.4217	0.7498	0.0039	0.488
C09: Agents acting on the renin–angiotensin system	Cutaneous melanoma	147.9214	0.811	0.0128	0.0897
C03: Diuretics	Malignant melanoma of the skin	72.632	0.7608	−0.0024	0.7479
C03: Diuretics	Cutaneous melanoma	95.8575	0.2658	−0.0042	0.7091
C08: Calcium channel blockers	Malignant melanoma of the skin	80.1599	0.7119	−0.004	0.5917
C08: Calcium channel blockers	Cutaneous melanoma	73.6709	0.9076	0.0005	0.9594
H03A: Thyroid preparations	Malignant melanoma of the skin	78.7658	0.9951	−0.0012	0.7899
H03A: Thyroid preparations	Cutaneous melanoma	93.6669	0.9278	0.0041	0.5452
C07: Beta blocking agents	Malignant melanoma of the skin	72.1946	0.0714	−0.0145	0.2593
C07: Beta blocking agents	Cutaneous melanoma	43.9047	0.8089	0.0022	0.8846
C10AA: HMG CoA reductase inhibitors	Malignant melanoma of the skin	61.2164	0.9802	0.005	0.3039
C10AA: HMG CoA reductase inhibitors	Cutaneous melanoma	65.969	0.9269	0.0045	0.4933
A10: Drugs used in diabetes	Malignant melanoma of the skin	34.0802	0.903	−0.012	0.1198
A10: Drugs used in diabetes	Cutaneous melanoma	44.8823	0.6014	0.0054	0.6097
N02A: Opioids	Malignant melanoma of the skin	1.5901	0.4516	−0.057	0.8508
N02A: Opioids	Cutaneous melanoma	3.0697	0.2155	0.4393	0.3356
C02: Antihypertensives	Malignant melanoma of the skin	0.2257	0.9733	0.1139	0.73
C02: Antihypertensives	Cutaneous melanoma	4.7457	0.1914	0.3436	0.6233
C01D: Vasodilators used in cardiac diseases	Malignant melanoma of the skin	0.7753	0.3786	NA	NA
C01D: Vasodilators used in cardiac diseases	Cutaneous melanoma	2.8943	0.0889	NA	NA
M01A: Anti-inflammatory and antirheumatic products, non-steroids	Malignant melanoma of the skin	6.121	0.2946	0.0214	0.7795
M01A: Anti-inflammatory and antirheumatic products, non-steroids	Cutaneous melanoma	3.8206	0.2815	0.011	0.9368
R03A: Adrenergics, inhalants	Malignant melanoma of the skin	44.5975	0.5726	−0.0029	0.7461
R03A: Adrenergics, inhalants	Cutaneous melanoma	55.5037	0.3806	−0.0067	0.5958
R03BA: Glucocorticoids	Malignant melanoma of the skin	15.7594	0.4699	0.0295	0.1124
R03BA: Glucocorticoids	Cutaneous melanoma	9.7239	0.9405	0.0045	0.8482
M05B: Drugs affecting bone structure and mineralization	Malignant melanoma of the skin	15.3649	0.1193	0.0615	0.1919
M05B: Drugs affecting bone structure and mineralization	Cutaneous melanoma	6.537	0.7683	0.0516	0.3026
S01E: Antiglaucoma preparations and miotics	Malignant melanoma of the skin	11.8261	0.3769	−0.0255	0.1875
S01E: Antiglaucoma preparations and miotics	Cutaneous melanoma	12.7507	0.3099	0.0562	0.0691
N02BA: Salicylic acid and derivatives	Malignant melanoma of the skin	6.0431	0.4184	0.0068	0.8252
N02BA: Salicylic acid and derivatives	Cutaneous melanoma	13.2612	0.1511	−0.027	0.5071
R06A: Antihistamines for systemic use	Malignant melanoma of the skin	8.6941	0.2754	0.0169	0.7685
R06A: Antihistamines for systemic use	Cutaneous melanoma	6.6625	0.4648	−0.1096	0.1144
A02B: Drugs for peptic ulcers and gastro-oesophageal reflux disease (GORD)	Malignant melanoma of the skin	7.6852	0.1038	0.1377	0.169
A02B: Drugs for peptic ulcers and gastro-oesophageal reflux disease (GORD)	Cutaneous melanoma	2.003	0.7352	0.0139	0.8744
N02BE: Anilides	Malignant melanoma of the skin	7.6643	0.1757	−0.0159	0.8456
N02BE: Anilides	Cutaneous melanoma	8.2897	0.2176	−0.0653	0.5015
N02C: Antimigraine preparations	Malignant melanoma of the skin	17.2075	0.142	0.0088	0.8618
N02C: Antimigraine preparations	Cutaneous melanoma	8.2093	0.7686	0.0561	0.3373
B01A: Antithrombotic agents	Malignant melanoma of the skin	5.4223	0.7116	0.0185	0.5226
B01A: Antithrombotic agents	Cutaneous melanoma	13.8993	0.3072	−0.0263	0.4107
L04: Immunosuppressants	Malignant melanoma of the skin	0.7494	0.3867	NA	NA
L04: Immunosuppressants	Cutaneous melanoma	0.2824	0.5951	NA	NA

**Table 4:** Steiger directionality test for each exposure–outcome pair.

Exposure	Outcome	Correct_Causal_Direction	Steiger_Pval
A02B: Drugs for peptic ulcers and gastro-oesophageal reflux disease (GORD)	Cutaneous melanoma	TRUE	1.02 × 10^−27^
A02B: Drugs for peptic ulcers and gastro-oesophageal reflux disease (GORD)	Malignant melanoma of the skin	TRUE	3.21 × 10^−25^
A10: Drugs used in diabetes	Cutaneous melanoma	TRUE	0
A10: Drugs used in diabetes	Malignant melanoma of the skin	TRUE	0
B01A: Antithrombotic agents	Cutaneous melanoma	TRUE	1.7 × 10^−115^
B01A: Antithrombotic agents	Malignant melanoma of the skin	TRUE	2.9 × 10^−101^
C01D: Vasodilators used in cardiac diseases	Cutaneous melanoma	TRUE	2.01 × 10^−17^
C01D: Vasodilators used in cardiac diseases	Malignant melanoma of the skin	TRUE	2.53 × 10^−18^
C02: Antihypertensives	Cutaneous melanoma	TRUE	1.45 × 10^−19^
C02: Antihypertensives	Malignant melanoma of the skin	TRUE	1.01 × 10^−22^
C03: Diuretics	Cutaneous melanoma	TRUE	0
C03: Diuretics	Malignant melanoma of the skin	TRUE	0
C07: Beta blocking agents	Cutaneous melanoma	TRUE	0
C07: Beta blocking agents	Malignant melanoma of the skin	TRUE	0
C08: Calcium channel blockers	Cutaneous melanoma	TRUE	0
C08: Calcium channel blockers	Malignant melanoma of the skin	TRUE	0
C09: Agents acting on the renin–angiotensin system	Cutaneous melanoma	TRUE	0
C09: Agents acting on the renin–angiotensin system	Malignant melanoma of the skin	TRUE	0
C10AA: HMG CoA reductase inhibitors	Cutaneous melanoma	TRUE	0
C10AA: HMG CoA reductase inhibitors	Malignant melanoma of the skin	TRUE	0
H03A: Thyroid preparations	Cutaneous melanoma	TRUE	0
H03A: Thyroid preparations	Malignant melanoma of the skin	TRUE	0
L04: Immunosuppressants	Cutaneous melanoma	TRUE	1.37 × 10^−73^
L04: Immunosuppressants	Malignant melanoma of the skin	TRUE	1.89 × 10^−67^
M01A: Anti-inflammatory and antirheumatic products, non-steroids	Cutaneous melanoma	TRUE	7.89 × 10^−29^
M01A: Anti-inflammatory and antirheumatic products, non-steroids	Malignant melanoma of the skin	TRUE	4.83 × 10^−32^
M05B: Drugs affecting bone structure and mineralization	Cutaneous melanoma	TRUE	3.74 × 10^−60^
M05B: Drugs affecting bone structure and mineralization	Malignant melanoma of the skin	TRUE	5.2 × 10^−55^
N02A: Opioids	Cutaneous melanoma	TRUE	1.12 × 10^−13^
N02A: Opioids	Malignant melanoma of the skin	TRUE	2.26 × 10^−15^
N02BA: Salicylic acid and derivatives	Cutaneous melanoma	TRUE	3.89 × 10^−69^
N02BA: Salicylic acid and derivatives	Malignant melanoma of the skin	TRUE	3.59 × 10^−62^
N02BE: Anilides	Cutaneous melanoma	TRUE	4.47 × 10^−46^
N02BE: Anilides	Malignant melanoma of the skin	TRUE	7.22 × 10^−39^
N02C: Antimigraine preparations	Cutaneous melanoma	TRUE	1.77 × 10^−88^
N02C: Antimigraine preparations	Malignant melanoma of the skin	TRUE	1.6 × 10^−79^
N06A: Antidepressants	Cutaneous melanoma	TRUE	3.16 × 10^−7^
N06A: Antidepressants	Malignant melanoma of the skin	TRUE	1.02 × 10^−6^
R03A: Adrenergics, inhalants	Cutaneous melanoma	TRUE	0
R03A: Adrenergics, inhalants	Malignant melanoma of the skin	TRUE	0
R03BA: Glucocorticoids	Cutaneous melanoma	TRUE	2.6 × 10^−192^
R03BA: Glucocorticoids	Malignant melanoma of the skin	TRUE	5.5 × 10^−174^
R06A: Antihistamines for systemic use	Cutaneous melanoma	TRUE	1.76 × 10^−45^
R06A: Antihistamines for systemic use	Malignant melanoma of the skin	TRUE	2.92 × 10^−42^
S01E: Antiglaucoma preparations and miotics	Cutaneous melanoma	TRUE	2.7 × 10^−103^
S01E: Antiglaucoma preparations and miotics	Malignant melanoma of the skin	TRUE	8.89 × 10^−96^

TRUE indicates that the inferred causal direction is from medication use to melanoma risk.

## Data Availability

All data generated or analyzed during this study are included in this published article and its Supplementary Information files. GWAS summary statistics for medication use were obtained from Wu et al. [20], and those for melanoma were obtained from the GWAS Catalog (GCST90041829) and FinnGen Release 12 (https://r12.finngen.fi/, (accessed on 20 September 2025)). The harmonized datasets and analysis scripts (including the code for TwoSampleMR v4.0.5) are available at https://github.com/phoenixwhy/my-project?tab=readme-ov-file#my-project (accessed on 20 September 2025).

## Supplementary Materials

The following supporting information can be downloaded at https://www.mdpi.com/article/10.3390/biomedicines13102477/s1. Table S1. Detailed information on the GWAS data; Table S2. Information on the instrument variable (IV). Table S3. Detailed SNPs and their statistical information. STROBE-MR checklist: Title: STROBE-MR checklist of recommended items to address in reports of Mendelian randomization studies. Supplementary Figure S1. (A–E) Sensitivity analyses for the association between genetically predicted use of agents acting on the renin–angiotensin system and malignant melanoma of skin (FinnGen). (A) Scatter plot of SNP effects; (B) forest plot of individual SNP estimates; (C) funnel plot for heterogeneity; (D) leave-one-out analysis; (E) radial plot for outlier estimation. These plots demonstrate the robustness of the causal estimate after outlier removal. Supplementary Figure S2. (A–E) Sensitivity analyses for the association between genetically predicted use of thyroid preparations and malignant melanoma of skin (FinnGen). (A) Scatter plot of SNP effects; (B) forest plot of individual SNP estimates; (C) funnel plot for heterogeneity; (D) leave-one-out analysis; (E) radial plot for outlier estimation. These plots demonstrate the consistency and robustness of the causal estimate after outlier removal. Supplementary Figure S3. (A–E) Sensitivity analyses for the association between genetically predicted use of drugs used in diabetes and malignant melanoma of skin (FinnGen). (A) Scatter plot of SNP effects; (B) forest plot of individual SNP estimates; (C) funnel plot for heterogeneity; (D) leave-one-out analysis; (E) radial plot for outlier estimation. These plots demonstrate the robustness of the causal estimate after outlier removal. Supplementary Figure S4. (A–D) Sensitivity analyses for the association between genetically predicted use of adrenergics, inhalants, and cutaneous melanoma (UK Biobank). (A) Scatter plot of SNP effects; (B) forest plot of individual SNP estimates; (C) funnel plot for heterogeneity; (D) leave-one-out analysis. These plots support the robustness of the results. Supplementary Figure S5. (A–D) Sensitivity analyses for the association between genetically predicted glucocorticoid use and cutaneous melanoma (UK Biobank). (A) Scatter plot of SNP effects; (B) forest plot of individual SNP estimates; (C) funnel plot for heterogeneity; (D) leave-one-out analysis. These plots support the robustness of the results. Supplementary Figure S6. (A–D) Sensitivity analyses for the association between genetically predicted opioid use and cutaneous melanoma (UK Biobank). (A) Scatter plot of SNP effects; (B) forest plot of individual SNP estimates; (C) funnel plot for heterogeneity; (D) leave-one-out analysis. Due to the limited number of SNPs, this result should be interpreted as exploratory.

## Abbreviations

The following abbreviations are used in this manuscript:

(GWASs)	Genome-Wide Association Studies
(MR)	Mendelian randomization
(DALYs)	Disability-adjusted life years
(NSAIDs)	Nonsteroidal anti-inflammatory medications
(COX)	Cyclooxygenase
(IVW)	Inverse variance weighted method
(WME)	Weighted median estimation
(GORD)	Gastro-esophageal reflux disease
