# Supplementary material for: Potential Risk of Cutaneous Melanoma Attributable to Medication Use: A Mendelian Randomization Approach
Source: Biomedicines. 2025 Oct 11;13(10):2477. doi: 10.3390/biomedicines13102477 (PMC12561804; doi:10.3390/biomedicines13102477)
Supplement: Supplementary file 1 [file biomedicines-13-02477-s001.zip › Supplementary material Tables.docx]

Supplementary Table S1. GWAS datasets for exposures and melanoma outcomes used in Mendelian randomization.

| **Trait** | **GWAS ID** | **Sample size（case/control）** |
| --- | --- | --- |
| cutaneous melanoma | GCST90041829 | 2,824/453,524 |
| Malignant melanoma of skin, excluding all cancers (controls excluding all cancers) | C3_MELANOMA_SKIN_EXALLC | 5,753/378,749 |
| Peptic ulcer and gastro-oesophageal reflux disease (GORD) drug use measurement | CST007922 | 132,367 |
| Drugs used in diabetes use measurement | GCST007923 | 132,367 |
| Antithrombotic agent use measurement | GCST007924 | 132,367 |
| Vasodilators used in cardiac diseases use measurement | GCST007925 | 132,367 |
| Antihypertensive use measurement | GCST007926 | 132,367 |
| Diuretic use measurement | GCST007927 | 132,367 |
| Beta blocking agent use measurement | GCST007928 | 132,367 |
| Calcium channel blocker use measurement | GCST007929 | 132,367 |
| Agents acting on the renin-angiotensin system use measurement | GCST007930 | 132,367 |
| HMG CoA reductase inhibitor use measurement | GCST007931 | 132,367 |
| Thyroid preparation use measurement | GCST007932 | 132,367 |
| Immunosuppressant use measurement | GCST007933 | 132,367 |
| Non-steroidal anti-inflammatory and antirheumatic product use measurement | GCST007934 | 132,367 |
| Drugs affecting bone structure and mineralization use measurement | GCST007935 | 132,367 |
| Opioid use measurement | GCST007936 | 132,367 |
| aspirin use measurement | GCST007937 | 132,367 |
| Anilide use measurement | GCST007938 | 132,367 |
| Antimigraine preparation use measurement | GCST007939 | 132,367 |
| Antidepressant use measurement | GCST007940 | 132,367 |
| Inhalant adrenergic use measurement | GCST007941 | 132,367 |
| Glucocorticoid use measurement | GCST007942 | 132,367 |
| Antihistamine use measurement | GCST007943 | 132,367 |
| Antiglaucoma preparations and miotics use measurement | GCST007944 | 132,367 |

Supplementary Table S2. Power calculations and minimum detectable odds ratios for each exposure–outcome pair.

| Exposure | Outcome | IVW_OR | Exposure_R2 | N | K | Power |
| --- | --- | --- | --- | --- | --- | --- |
| C09: Agents acting on the renin-angiotensin system | Malignant melanoma of skin | 1.094448 | 0.069341 | 132367 | 0.014962 | 19.52% |
| C09: Agents acting on the renin-angiotensin system | cutaneous melanoma | 1.081218 | 0.070271 | 132367 | 0.006188 | 9.42% |
| C03: Diuretics | Malignant melanoma of skin | 1.085859 | 0.037596 | 132367 | 0.014962 | 11.37% |
| C03: Diuretics | cutaneous melanoma | 1.048642 | 0.037836 | 132367 | 0.006188 | 5.84% |
| C08: Calcium channel blockers | Malignant melanoma of skin | 1.079122 | 0.037178 | 132367 | 0.014962 | 10.33% |
| C08: Calcium channel blockers | cutaneous melanoma | 1.031136 | 0.037353 | 132367 | 0.006188 | 5.34% |
| H03A: Thyroid preparations | Malignant melanoma of skin | 0.949967 | 0.076403 | 132367 | 0.014962 | 9.38% |
| H03A: Thyroid preparations | cutaneous melanoma | 0.955905 | 0.075829 | 132367 | 0.006188 | 6.39% |
| C07: Beta blocking agents | Malignant melanoma of skin | 1.062894 | 0.018659 | 132367 | 0.014962 | 6.66% |
| C07: Beta blocking agents | cutaneous melanoma | 1.037248 | 0.019001 | 132367 | 0.006188 | 5.25% |
| C10AA: HMG CoA reductase inhibitors | Malignant melanoma of skin | 1.09269 | 0.064209 | 132367 | 0.014962 | 17.91% |
| C10AA: HMG CoA reductase inhibitors | cutaneous melanoma | 1.075208 | 0.064209 | 132367 | 0.006188 | 8.45% |
| A10: Drugs used in diabetes | Malignant melanoma of skin | 0.968743 | 0.027147 | 132367 | 0.014962 | 5.60% |
| A10: Drugs used in diabetes | cutaneous melanoma | 1.014941 | 0.026747 | 132367 | 0.006188 | 5.06% |
| N02A: Opioids | Malignant melanoma of skin | 0.849037 | 0.000795 | 132367 | 0.014962 | 5.41% |
| N02A: Opioids | cutaneous melanoma | 0.49339 | 0.000795 | 132367 | 0.006188 | 6.94% |
| C02: Antihypertensives | Malignant melanoma of skin | 0.970176 | 0.001006 | 132367 | 0.014962 | 5.02% |
| C02: Antihypertensives | cutaneous melanoma | 0.892373 | 0.001006 | 132367 | 0.006188 | 5.11% |
| C01D: Vasodilators used in cardiac diseases | Malignant melanoma of skin | 1.015807 | 0.000859 | 132367 | 0.014962 | 5.00% |
| C01D: Vasodilators used in cardiac diseases | cutaneous melanoma | 0.895232 | 0.000859 | 132367 | 0.006188 | 5.09% |
| M01A: Antiinflammatroy and antirheumatic products, non-steroids | Malignant melanoma of skin | 0.975419 | 0.001736 | 132367 | 0.014962 | 5.02% |
| M01A: Antiinflammatroy and antirheumatic products, non-steroids | cutaneous melanoma | 1.008411 | 0.001736 | 132367 | 0.006188 | 5.00% |
| R03A: Adrenergics, inhalants | Malignant melanoma of skin | 0.97075 | 0.026426 | 132367 | 0.014962 | 5.51% |
| R03A: Adrenergics, inhalants | cutaneous melanoma | 0.867601 | 0.025587 | 132367 | 0.006188 | 9.28% |
| R03BA: Glucocorticoids | Malignant melanoma of skin | 0.995398 | 0.009634 | 132367 | 0.014962 | 5.00% |
| R03BA: Glucocorticoids | cutaneous melanoma | 0.832485 | 0.009634 | 132367 | 0.006188 | 7.56% |
| M05B: Drugs affecting bone structure and mineralization | Malignant melanoma of skin | 1.032022 | 0.003063 | 132367 | 0.014962 | 5.07% |
| M05B: Drugs affecting bone structure and mineralization | cutaneous melanoma | 0.888485 | 0.003063 | 132367 | 0.006188 | 5.36% |
| S01E: Antiglaucoma preparations and miotics | Malignant melanoma of skin | 1.04732 | 0.00559 | 132367 | 0.014962 | 5.28% |
| S01E: Antiglaucoma preparations and miotics | cutaneous melanoma | 1.023586 | 0.00559 | 132367 | 0.006188 | 5.03% |
| N02BA: Salicylic acid and derivatives | Malignant melanoma of skin | 1.089555 | 0.003625 | 132367 | 0.014962 | 5.65% |
| N02BA: Salicylic acid and derivatives | cutaneous melanoma | 0.93765 | 0.003625 | 132367 | 0.006188 | 5.13% |
| R06A: Antihistamines for systemic use | Malignant melanoma of skin | 1.023175 | 0.002304 | 132367 | 0.014962 | 5.03% |
| R06A: Antihistamines for systemic use | cutaneous melanoma | 0.959539 | 0.002304 | 132367 | 0.006188 | 5.04% |
| A02B: Drugs for peptic ulcer and gastro-oesophageal reflux disease (GORD) | Malignant melanoma of skin | 1.098685 | 0.001383 | 132367 | 0.014962 | 5.30% |
| A02B: Drugs for peptic ulcer and gastro-oesophageal reflux disease (GORD) | cutaneous melanoma | 0.664746 | 0.001383 | 132367 | 0.006188 | 6.47% |
| N02BE: Anilides | Malignant melanoma of skin | 0.706373 | 0.002411 | 132367 | 0.014962 | 9.81% |
| N02BE: Anilides | cutaneous melanoma | 0.746263 | 0.002411 | 132367 | 0.006188 | 6.47% |
| N02C: Antimigraine preparations | Malignant melanoma of skin | 1.038676 | 0.004422 | 132367 | 0.014962 | 5.15% |
| N02C: Antimigraine preparations | cutaneous melanoma | 1.038161 | 0.004422 | 132367 | 0.006188 | 5.06% |
| B01A: Antithrombotic agents | Malignant melanoma of skin | 1.066197 | 0.005879 | 132367 | 0.014962 | 5.58% |
| B01A: Antithrombotic agents | cutaneous melanoma | 0.983618 | 0.005879 | 132367 | 0.006188 | 5.01% |
| L04: Immunosuppressants | Malignant melanoma of skin | 0.932516 | 0.003462 | 132367 | 0.014962 | 5.35% |
| L04: Immunosuppressants | cutaneous melanoma | 0.922797 | 0.003462 | 132367 | 0.006188 | 5.19% |

K = proportion of cases, N = sample size.

Supplementary Table S3. List of outlier SNPs removed in sensitivity analyses.

| SNP | method | iteration | reason | exposure | outcome |  |
| --- | --- | --- | --- | --- | --- | --- |
| rs11556924 | RadialMR | 1 | Double outlier (IVW or Egger) | C09: Agents acting on the renin-angiotensin system | Malignant melanoma of skin | |
| rs1290786 | RadialMR | 1 | Double outlier (IVW or Egger) | C09: Agents acting on the renin-angiotensin system | Malignant melanoma of skin | |
| rs2252867 | RadialMR | 1 | Double outlier (IVW or Egger) | C09: Agents acting on the renin-angiotensin system | Malignant melanoma of skin | |
| rs268263 | RadialMR | 1 | Double outlier (IVW or Egger) | C09: Agents acting on the renin-angiotensin system | Malignant melanoma of skin | |
| rs28520411 | RadialMR | 1 | Double outlier (IVW or Egger) | C09: Agents acting on the renin-angiotensin system | Malignant melanoma of skin | |
| rs36071027 | RadialMR | 1 | Double outlier (IVW or Egger) | C09: Agents acting on the renin-angiotensin system | Malignant melanoma of skin | |
| rs57139556 | RadialMR | 1 | Double outlier (IVW or Egger) | C09: Agents acting on the renin-angiotensin system | Malignant melanoma of skin | |
| rs57541197 | RadialMR | 1 | Double outlier (IVW or Egger) | C09: Agents acting on the renin-angiotensin system | Malignant melanoma of skin | |
| rs6021247 | RadialMR | 1 | Double outlier (IVW or Egger) | C09: Agents acting on the renin-angiotensin system | Malignant melanoma of skin | |
| rs72681869 | RadialMR | 1 | Double outlier (IVW or Egger) | C09: Agents acting on the renin-angiotensin system | Malignant melanoma of skin | |
| rs7412 | RadialMR | 1 | Double outlier (IVW or Egger) | C09: Agents acting on the renin-angiotensin system | Malignant melanoma of skin | |
| rs964184 | RadialMR | 1 | Double outlier (IVW or Egger) | C09: Agents acting on the renin-angiotensin system | Malignant melanoma of skin | |
| rs9897348 | RadialMR | 1 | Double outlier (IVW or Egger) | C09: Agents acting on the renin-angiotensin system | Malignant melanoma of skin | |
| rs11105352 | RadialMR | 1 | Double outlier (IVW or Egger) | C03: Diuretics | Malignant melanoma of skin | |
| rs12428857 | RadialMR | 1 | Double outlier (IVW or Egger) | C03: Diuretics | Malignant melanoma of skin | |
| rs1407256 | RadialMR | 1 | Double outlier (IVW or Egger) | C03: Diuretics | Malignant melanoma of skin | |
| rs17080089 | RadialMR | 1 | Double outlier (IVW or Egger) | C03: Diuretics | Malignant melanoma of skin | |
| rs2421647 | RadialMR | 1 | Double outlier (IVW or Egger) | C03: Diuretics | Malignant melanoma of skin | |
| rs268263 | RadialMR | 1 | Double outlier (IVW or Egger) | C03: Diuretics | Malignant melanoma of skin | |
| rs4537305 | RadialMR | 1 | Double outlier (IVW or Egger) | C03: Diuretics | Malignant melanoma of skin | |
| rs5417 | RadialMR | 1 | Double outlier (IVW or Egger) | C03: Diuretics | Malignant melanoma of skin | |
| rs6021247 | RadialMR | 1 | Double outlier (IVW or Egger) | C03: Diuretics | Malignant melanoma of skin | |
| rs72681869 | RadialMR | 1 | Double outlier (IVW or Egger) | C03: Diuretics | Malignant melanoma of skin | |
| rs72910057 | RadialMR | 1 | Double outlier (IVW or Egger) | C03: Diuretics | Malignant melanoma of skin | |
| rs7599224 | RadialMR | 1 | Double outlier (IVW or Egger) | C03: Diuretics | Malignant melanoma of skin | |
| rs891556 | RadialMR | 1 | Double outlier (IVW or Egger) | C08: Calcium channel blockers | Malignant melanoma of skin | |
| rs11105352 | RadialMR | 1 | Double outlier (IVW or Egger) | C08: Calcium channel blockers | Malignant melanoma of skin | |
| rs11556924 | RadialMR | 1 | Double outlier (IVW or Egger) | C08: Calcium channel blockers | Malignant melanoma of skin | |
| rs2276838 | RadialMR | 1 | Double outlier (IVW or Egger) | C08: Calcium channel blockers | Malignant melanoma of skin | |
| rs3740393 | RadialMR | 1 | Double outlier (IVW or Egger) | C08: Calcium channel blockers | Malignant melanoma of skin | |
| rs6021247 | RadialMR | 1 | Double outlier (IVW or Egger) | C08: Calcium channel blockers | Malignant melanoma of skin | |
| rs60370741 | RadialMR | 1 | Double outlier (IVW or Egger) | C08: Calcium channel blockers | Malignant melanoma of skin | |
| rs6078002 | RadialMR | 1 | Double outlier (IVW or Egger) | C08: Calcium channel blockers | Malignant melanoma of skin | |
| rs62434123 | RadialMR | 1 | Double outlier (IVW or Egger) | C08: Calcium channel blockers | Malignant melanoma of skin | |
| rs72681869 | RadialMR | 1 | Double outlier (IVW or Egger) | C08: Calcium channel blockers | Malignant melanoma of skin | |
| rs7412 | RadialMR | 1 | Double outlier (IVW or Egger) | C08: Calcium channel blockers | Malignant melanoma of skin | |
| rs7754251 | RadialMR | 1 | Double outlier (IVW or Egger) | H03A: Thyroid preparations | Malignant melanoma of skin | |
| rs10836367 | RadialMR | 1 | Double outlier (IVW or Egger) | H03A: Thyroid preparations | Malignant melanoma of skin | |
| rs11784499 | RadialMR | 1 | Double outlier (IVW or Egger) | H03A: Thyroid preparations | Malignant melanoma of skin | |
| rs12325861 | RadialMR | 1 | Double outlier (IVW or Egger) | H03A: Thyroid preparations | Malignant melanoma of skin | |
| rs12634152 | RadialMR | 1 | Double outlier (IVW or Egger) | H03A: Thyroid preparations | Malignant melanoma of skin | |
| rs12742404 | RadialMR | 1 | Double outlier (IVW or Egger) | H03A: Thyroid preparations | Malignant melanoma of skin | |
| rs13076468 | RadialMR | 1 | Double outlier (IVW or Egger) | H03A: Thyroid preparations | Malignant melanoma of skin | |
| rs2111485 | RadialMR | 1 | Double outlier (IVW or Egger) | H03A: Thyroid preparations | Malignant melanoma of skin | |
| rs2858483 | RadialMR | 1 | Double outlier (IVW or Egger) | H03A: Thyroid preparations | Malignant melanoma of skin | |
| rs366327 | RadialMR | 1 | Double outlier (IVW or Egger) | H03A: Thyroid preparations | Malignant melanoma of skin | |
| rs6914622 | RadialMR | 1 | Double outlier (IVW or Egger) | H03A: Thyroid preparations | Malignant melanoma of skin | |
| rs7254729 | RadialMR | 1 | Double outlier (IVW or Egger) | H03A: Thyroid preparations | Malignant melanoma of skin | |
| rs72977594 | RadialMR | 1 | Double outlier (IVW or Egger) | H03A: Thyroid preparations | Malignant melanoma of skin | |
| rs7831557 | RadialMR | 1 | Double outlier (IVW or Egger) | H03A: Thyroid preparations | Malignant melanoma of skin | |
| rs9291444 | RadialMR | 1 | Double outlier (IVW or Egger) | H03A: Thyroid preparations | Malignant melanoma of skin | |
| rs2982521 | RadialMR | 1 | Double outlier (IVW or Egger) | C10AA: HMG CoA reductase inhibitors | Malignant melanoma of skin | |
| rs10804330 | RadialMR | 1 | Double outlier (IVW or Egger) | C10AA: HMG CoA reductase inhibitors | Malignant melanoma of skin | |
| rs11057830 | RadialMR | 1 | Double outlier (IVW or Egger) | C10AA: HMG CoA reductase inhibitors | Malignant melanoma of skin | |
| rs2917677 | RadialMR | 1 | Double outlier (IVW or Egger) | C10AA: HMG CoA reductase inhibitors | Malignant melanoma of skin | |
| rs35430985 | RadialMR | 1 | Double outlier (IVW or Egger) | C10AA: HMG CoA reductase inhibitors | Malignant melanoma of skin | |
| rs7412 | RadialMR | 1 | Double outlier (IVW or Egger) | C10AA: HMG CoA reductase inhibitors | Malignant melanoma of skin | |
| rs8126001 | RadialMR | 1 | Double outlier (IVW or Egger) | C10AA: HMG CoA reductase inhibitors | Malignant melanoma of skin | |
| rs964184 | RadialMR | 1 | Double outlier (IVW or Egger) | C10AA: HMG CoA reductase inhibitors | Malignant melanoma of skin | |
| rs116782923 | RadialMR | 1 | Double outlier (IVW or Egger) | A10: Drugs used in diabetes | Malignant melanoma of skin | |
| rs145510090 | RadialMR | 1 | Double outlier (IVW or Egger) | A10: Drugs used in diabetes | Malignant melanoma of skin | |
| rs2293476 | RadialMR | 1 | Double outlier (IVW or Egger) | A10: Drugs used in diabetes | Malignant melanoma of skin | |
| rs2723063 | RadialMR | 1 | Double outlier (IVW or Egger) | A10: Drugs used in diabetes | Malignant melanoma of skin | |
| rs2972155 | RadialMR | 1 | Double outlier (IVW or Egger) | A10: Drugs used in diabetes | Malignant melanoma of skin | |
| rs3802177 | RadialMR | 1 | Double outlier (IVW or Egger) | A10: Drugs used in diabetes | Malignant melanoma of skin | |
| rs464605 | RadialMR | 1 | Double outlier (IVW or Egger) | A10: Drugs used in diabetes | Malignant melanoma of skin | |
| rs7766070 | RadialMR | 1 | Double outlier (IVW or Egger) | A10: Drugs used in diabetes | Malignant melanoma of skin | |
| rs9854769 | RadialMR | 1 | Double outlier (IVW or Egger) | A10: Drugs used in diabetes | Malignant melanoma of skin | |
| rs9957264 | RadialMR | 1 | Double outlier (IVW or Egger) | A10: Drugs used in diabetes | Malignant melanoma of skin | |
| rs12123821 | RadialMR | 1 | Double outlier (IVW or Egger) | R03BA: Glucocorticoids | Malignant melanoma of skin | |
| rs1504215 | RadialMR | 1 | Double outlier (IVW or Egger) | R03BA: Glucocorticoids | Malignant melanoma of skin | |
| rs12123821 | RadialMR | 1 | Double outlier (IVW or Egger) | R03A: Adrenergics, inhalants | Malignant melanoma of skin | |
| rs12788104 | RadialMR | 1 | Double outlier (IVW or Egger) | R03A: Adrenergics, inhalants | Malignant melanoma of skin | |
| rs1504215 | RadialMR | 1 | Double outlier (IVW or Egger) | R03A: Adrenergics, inhalants | Malignant melanoma of skin | |
| rs2289790 | RadialMR | 1 | Double outlier (IVW or Egger) | R03A: Adrenergics, inhalants | Malignant melanoma of skin | |
| rs2548992 | RadialMR | 1 | Double outlier (IVW or Egger) | R03A: Adrenergics, inhalants | Malignant melanoma of skin | |
| rs60946162 | RadialMR | 1 | Double outlier (IVW or Egger) | R03A: Adrenergics, inhalants | Malignant melanoma of skin | |
| rs6802894 | RadialMR | 1 | Double outlier (IVW or Egger) | R03A: Adrenergics, inhalants | Malignant melanoma of skin | |
| rs7992229 | RadialMR | 1 | Double outlier (IVW or Egger) | R03A: Adrenergics, inhalants | Malignant melanoma of skin | |
| rs1360589 | RadialMR | 1 | Double outlier (IVW or Egger) | S01E: Antiglaucoma preparations and miotics | Malignant melanoma of skin | |
| rs34186918 | RadialMR | 1 | Double outlier (IVW or Egger) | S01E: Antiglaucoma preparations and miotics | Malignant melanoma of skin | |
| rs28601761 | RadialMR | 1 | Double outlier (IVW or Egger) | N02BA: Salicylic acid and derivatives | Malignant melanoma of skin | |
| rs7412 | RadialMR | 1 | Double outlier (IVW or Egger) | N02BA: Salicylic acid and derivatives | Malignant melanoma of skin | |
| rs964184 | RadialMR | 1 | Double outlier (IVW or Egger) | N02BA: Salicylic acid and derivatives | Malignant melanoma of skin | |
| rs28601761 | RadialMR | 1 | Double outlier (IVW or Egger) | B01A: Antithrombotic agents | Malignant melanoma of skin | |
| rs56214516 | RadialMR | 1 | Double outlier (IVW or Egger) | B01A: Antithrombotic agents | Malignant melanoma of skin | |
| rs7412 | RadialMR | 1 | Double outlier (IVW or Egger) | B01A: Antithrombotic agents | Malignant melanoma of skin | |
| rs964184 | RadialMR | 1 | Double outlier (IVW or Egger) | B01A: Antithrombotic agents | Malignant melanoma of skin | |
| rs17652520 | RadialMR | 1 | Double outlier (IVW or Egger) | N02BE: Anilides | Malignant melanoma of skin | |
| rs12634152 | RadialMR | 1 | Double outlier (IVW or Egger) | H03A: Thyroid preparations | cutaneous melanoma | |
| rs145268310 | RadialMR | 1 | Double outlier (IVW or Egger) | H03A: Thyroid preparations | cutaneous melanoma | |
| rs1724088 | RadialMR | 1 | Double outlier (IVW or Egger) | H03A: Thyroid preparations | cutaneous melanoma | |
| rs1872691 | RadialMR | 1 | Double outlier (IVW or Egger) | H03A: Thyroid preparations | cutaneous melanoma | |
| rs2111485 | RadialMR | 1 | Double outlier (IVW or Egger) | H03A: Thyroid preparations | cutaneous melanoma | |
| rs2274780 | RadialMR | 1 | Double outlier (IVW or Egger) | H03A: Thyroid preparations | cutaneous melanoma | |
| rs2856698 | RadialMR | 1 | Double outlier (IVW or Egger) | H03A: Thyroid preparations | cutaneous melanoma | |
| rs2969903 | RadialMR | 1 | Double outlier (IVW or Egger) | H03A: Thyroid preparations | cutaneous melanoma | |
| rs35776863 | RadialMR | 1 | Double outlier (IVW or Egger) | H03A: Thyroid preparations | cutaneous melanoma | |
| rs4688013 | RadialMR | 1 | Double outlier (IVW or Egger) | H03A: Thyroid preparations | cutaneous melanoma | |
| rs4915076 | RadialMR | 1 | Double outlier (IVW or Egger) | H03A: Thyroid preparations | cutaneous melanoma | |
| rs56249713 | RadialMR | 1 | Double outlier (IVW or Egger) | H03A: Thyroid preparations | cutaneous melanoma | |
| rs61776678 | RadialMR | 1 | Double outlier (IVW or Egger) | H03A: Thyroid preparations | cutaneous melanoma | |
| rs11105352 | RadialMR | 1 | Double outlier (IVW or Egger) | C08: Calcium channel blockers | cutaneous melanoma | |
| rs17608766 | RadialMR | 1 | Double outlier (IVW or Egger) | C08: Calcium channel blockers | cutaneous melanoma | |
| rs1894400 | RadialMR | 1 | Double outlier (IVW or Egger) | C08: Calcium channel blockers | cutaneous melanoma | |
| rs2341599 | RadialMR | 1 | Double outlier (IVW or Egger) | C08: Calcium channel blockers | cutaneous melanoma | |
| rs460879 | RadialMR | 1 | Double outlier (IVW or Egger) | C08: Calcium channel blockers | cutaneous melanoma | |
| rs62434123 | RadialMR | 1 | Double outlier (IVW or Egger) | C08: Calcium channel blockers | cutaneous melanoma | |
| rs6271 | RadialMR | 1 | Double outlier (IVW or Egger) | C08: Calcium channel blockers | cutaneous melanoma | |
| rs7174222 | RadialMR | 1 | Double outlier (IVW or Egger) | C08: Calcium channel blockers | cutaneous melanoma | |
| rs116881820 | RadialMR | 1 | Double outlier (IVW or Egger) | C10AA: HMG CoA reductase inhibitors | cutaneous melanoma | |
| rs11743303 | RadialMR | 1 | Double outlier (IVW or Egger) | C10AA: HMG CoA reductase inhibitors | cutaneous melanoma | |
| rs12740374 | RadialMR | 1 | Double outlier (IVW or Egger) | C10AA: HMG CoA reductase inhibitors | cutaneous melanoma | |
| rs221907 | RadialMR | 1 | Double outlier (IVW or Egger) | C10AA: HMG CoA reductase inhibitors | cutaneous melanoma | |
| rs2917677 | RadialMR | 1 | Double outlier (IVW or Egger) | C10AA: HMG CoA reductase inhibitors | cutaneous melanoma | |
| rs3802932 | RadialMR | 1 | Double outlier (IVW or Egger) | C10AA: HMG CoA reductase inhibitors | cutaneous melanoma | |
| rs72837687 | RadialMR | 1 | Double outlier (IVW or Egger) | C10AA: HMG CoA reductase inhibitors | cutaneous melanoma | |
| rs73013176 | RadialMR | 1 | Double outlier (IVW or Egger) | C10AA: HMG CoA reductase inhibitors | cutaneous melanoma | |
| rs76895963 | RadialMR | 1 | Double outlier (IVW or Egger) | C10AA: HMG CoA reductase inhibitors | cutaneous melanoma | |
| rs8126001 | RadialMR | 1 | Double outlier (IVW or Egger) | C10AA: HMG CoA reductase inhibitors | cutaneous melanoma | |
| rs11874 | RadialMR | 1 | Double outlier (IVW or Egger) | C09: Agents acting on the renin-angiotensin system | cutaneous melanoma | |
| rs12549801 | RadialMR | 1 | Double outlier (IVW or Egger) | C09: Agents acting on the renin-angiotensin system | cutaneous melanoma | |
| rs164749 | RadialMR | 1 | Double outlier (IVW or Egger) | C09: Agents acting on the renin-angiotensin system | cutaneous melanoma | |
| rs1894400 | RadialMR | 1 | Double outlier (IVW or Egger) | C09: Agents acting on the renin-angiotensin system | cutaneous melanoma | |
| rs2236295 | RadialMR | 1 | Double outlier (IVW or Egger) | C09: Agents acting on the renin-angiotensin system | cutaneous melanoma | |
| rs2493298 | RadialMR | 1 | Double outlier (IVW or Egger) | C09: Agents acting on the renin-angiotensin system | cutaneous melanoma | |
| rs2656918 | RadialMR | 1 | Double outlier (IVW or Egger) | C09: Agents acting on the renin-angiotensin system | cutaneous melanoma | |
| rs3802932 | RadialMR | 1 | Double outlier (IVW or Egger) | C09: Agents acting on the renin-angiotensin system | cutaneous melanoma | |
| rs3857599 | RadialMR | 1 | Double outlier (IVW or Egger) | C09: Agents acting on the renin-angiotensin system | cutaneous melanoma | |
| rs57139556 | RadialMR | 1 | Double outlier (IVW or Egger) | C09: Agents acting on the renin-angiotensin system | cutaneous melanoma | |
| rs6068117 | RadialMR | 1 | Double outlier (IVW or Egger) | C09: Agents acting on the renin-angiotensin system | cutaneous melanoma | |
| rs7149242 | RadialMR | 1 | Double outlier (IVW or Egger) | C09: Agents acting on the renin-angiotensin system | cutaneous melanoma | |
| rs7174222 | RadialMR | 1 | Double outlier (IVW or Egger) | C09: Agents acting on the renin-angiotensin system | cutaneous melanoma | |
| rs751984 | RadialMR | 1 | Double outlier (IVW or Egger) | C09: Agents acting on the renin-angiotensin system | cutaneous melanoma | |
| rs77463690 | RadialMR | 1 | Double outlier (IVW or Egger) | C09: Agents acting on the renin-angiotensin system | cutaneous melanoma | |
| rs9844972 | RadialMR | 1 | Double outlier (IVW or Egger) | C09: Agents acting on the renin-angiotensin system | cutaneous melanoma | |
| rs12522598 | RadialMR | 1 | Double outlier (IVW or Egger) | M01A: Antiinflammatroy and antirheumatic products, non-steroids | cutaneous melanoma | |
| rs56166763 | RadialMR | 1 | Double outlier (IVW or Egger) | M01A: Antiinflammatroy and antirheumatic products, non-steroids | cutaneous melanoma | |
| rs10764331 | RadialMR | 1 | Double outlier (IVW or Egger) | C03: Diuretics | cutaneous melanoma | |
| rs11105352 | RadialMR | 1 | Double outlier (IVW or Egger) | C03: Diuretics | cutaneous melanoma | |
| rs17080089 | RadialMR | 1 | Double outlier (IVW or Egger) | C03: Diuretics | cutaneous melanoma | |
| rs2999159 | RadialMR | 1 | Double outlier (IVW or Egger) | C03: Diuretics | cutaneous melanoma | |
| rs557675 | RadialMR | 1 | Double outlier (IVW or Egger) | C03: Diuretics | cutaneous melanoma | |
| rs6536076 | RadialMR | 1 | Double outlier (IVW or Egger) | C03: Diuretics | cutaneous melanoma | |
| rs7174222 | RadialMR | 1 | Double outlier (IVW or Egger) | C03: Diuretics | cutaneous melanoma | |
| rs1496653 | RadialMR | 1 | Double outlier (IVW or Egger) | A10: Drugs used in diabetes | cutaneous melanoma | |
| rs1974619 | RadialMR | 1 | Double outlier (IVW or Egger) | A10: Drugs used in diabetes | cutaneous melanoma | |
| rs2258238 | RadialMR | 1 | Double outlier (IVW or Egger) | A10: Drugs used in diabetes | cutaneous melanoma | |
| rs3217792 | RadialMR | 1 | Double outlier (IVW or Egger) | A10: Drugs used in diabetes | cutaneous melanoma | |
| rs4715207 | RadialMR | 1 | Double outlier (IVW or Egger) | A10: Drugs used in diabetes | cutaneous melanoma | |
| rs5213 | RadialMR | 1 | Double outlier (IVW or Egger) | A10: Drugs used in diabetes | cutaneous melanoma | |
| rs668799 | RadialMR | 1 | Double outlier (IVW or Egger) | A10: Drugs used in diabetes | cutaneous melanoma | |
| rs258317 | RadialMR | 1 | Double outlier (IVW or Egger) | C07: Beta blocking agents | cutaneous melanoma | |
| rs55988870 | RadialMR | 1 | Double outlier (IVW or Egger) | C07: Beta blocking agents | cutaneous melanoma | |
| rs664485 | RadialMR | 1 | Double outlier (IVW or Egger) | C07: Beta blocking agents | cutaneous melanoma | |
| rs778124 | RadialMR | 1 | Double outlier (IVW or Egger) | C07: Beta blocking agents | cutaneous melanoma | |
| rs111867185 | RadialMR | 1 | Double outlier (IVW or Egger) | S01E: Antiglaucoma preparations and miotics | cutaneous melanoma | |
| rs34186918 | RadialMR | 1 | Double outlier (IVW or Egger) | S01E: Antiglaucoma preparations and miotics | cutaneous melanoma | |
