# Supplementary figures and images for: Potential Risk of Cutaneous Melanoma Attributable to Medication Use: A Mendelian Randomization Approach
Source: Biomedicines. 2025 Oct 11;13(10):2477. doi: 10.3390/biomedicines13102477 (PMC12561804; doi:10.3390/biomedicines13102477)

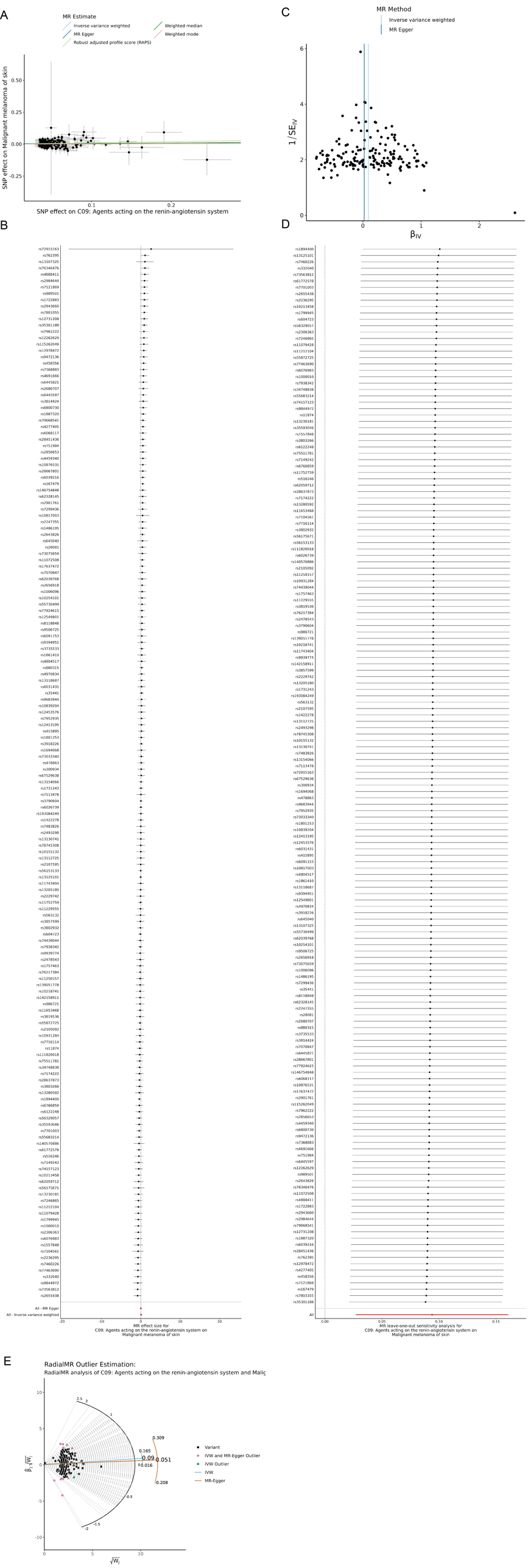

Supplement: Supplementary file 1 [file biomedicines-13-02477-s001.zip › Figure S1.jpg]

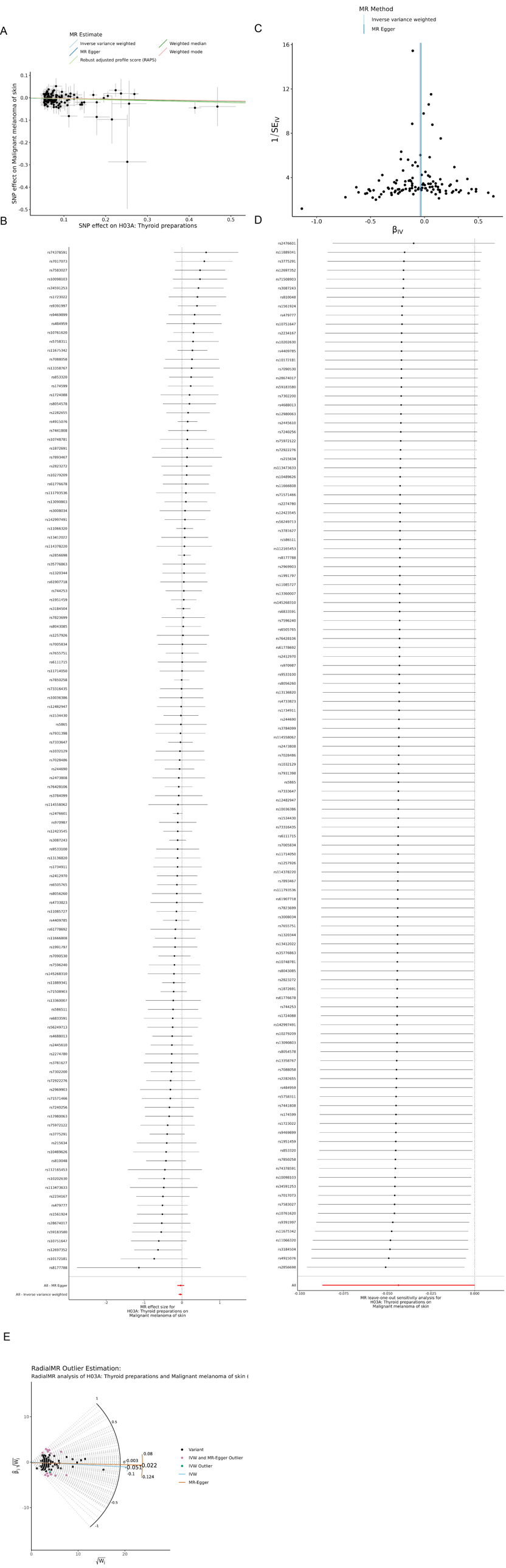

Supplement: Supplementary file 1 [file biomedicines-13-02477-s001.zip › Figure S2.jpg]

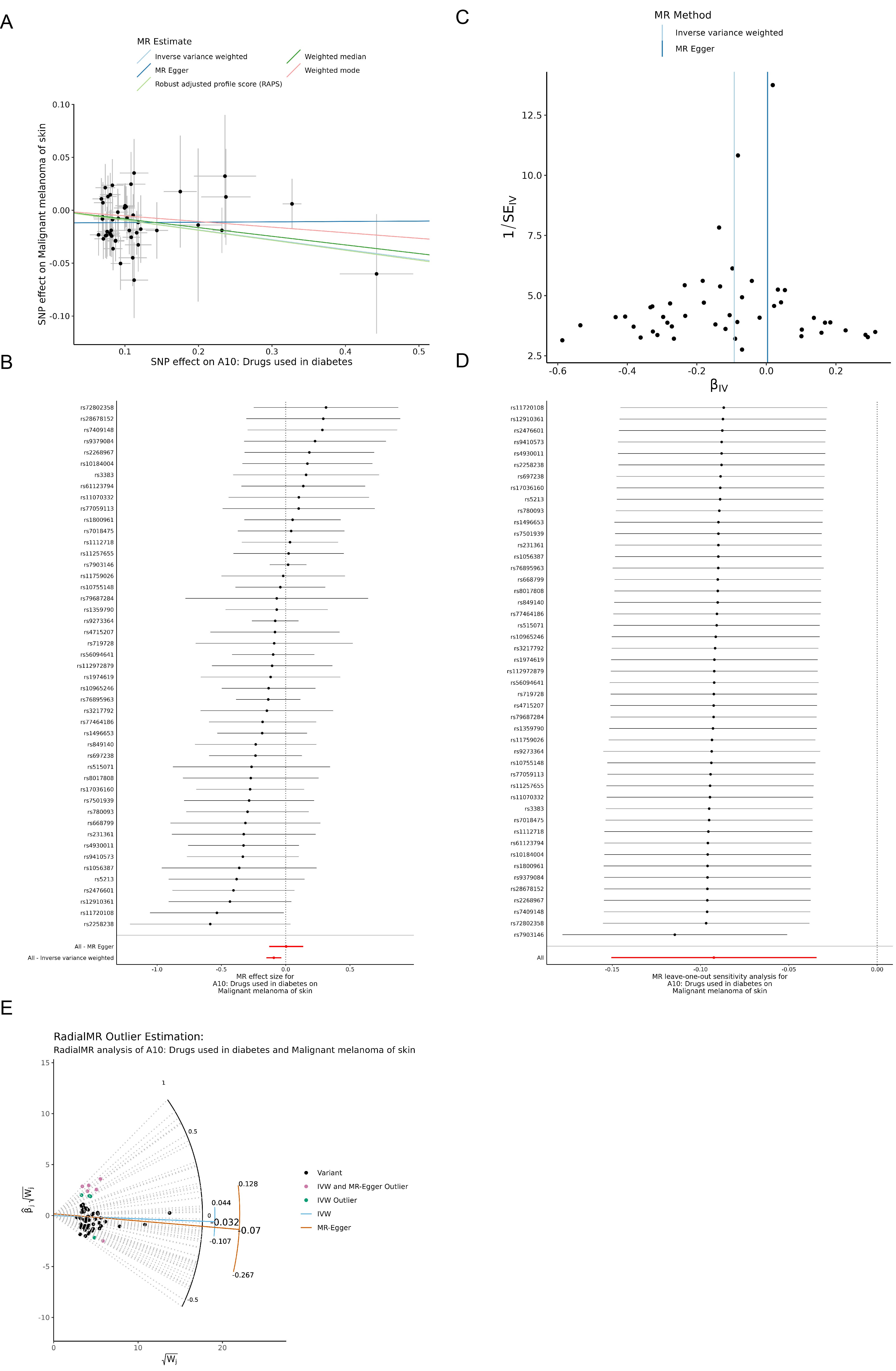

Supplement: Supplementary file 1 [file biomedicines-13-02477-s001.zip › Figure S3.jpg]

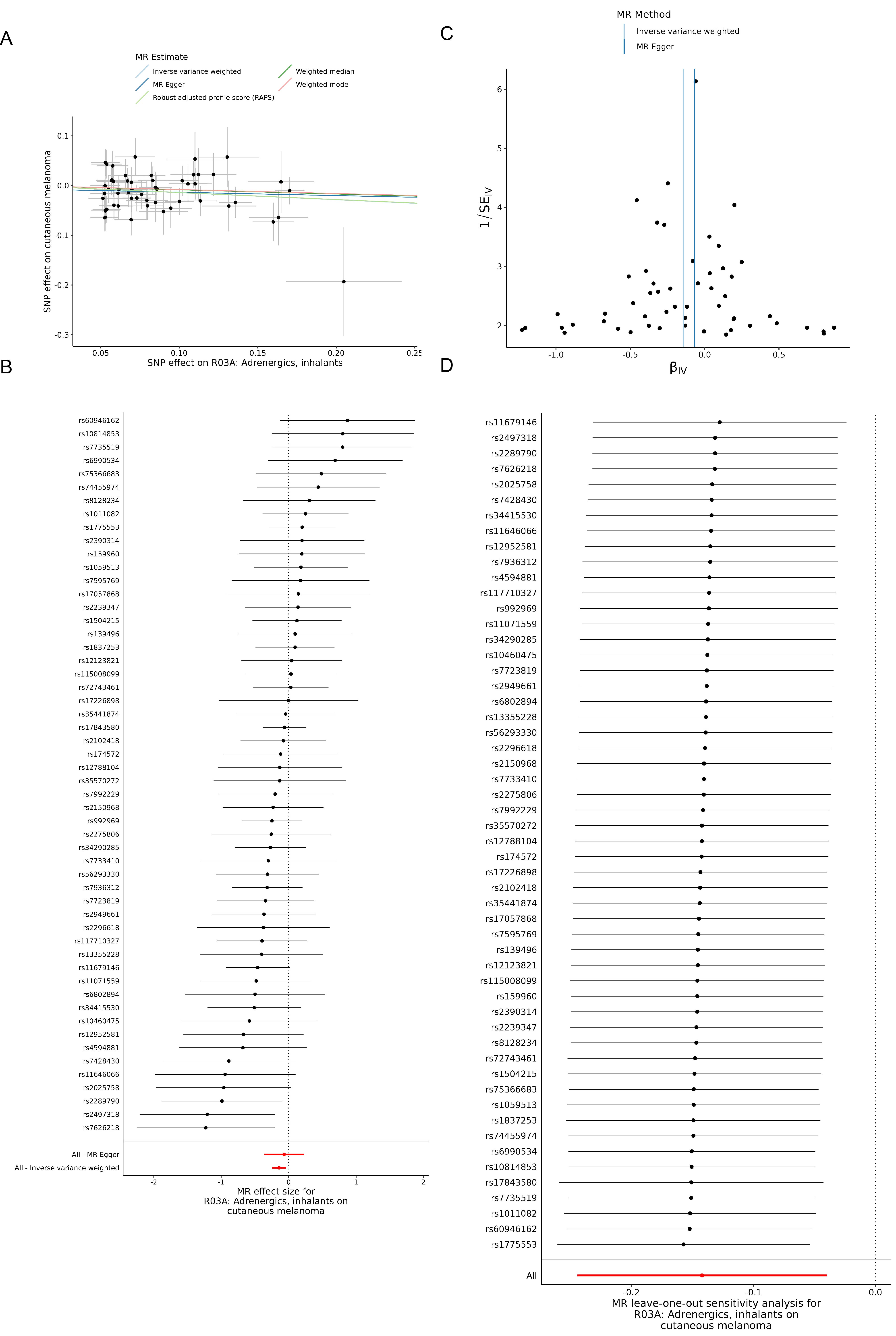

Supplement: Supplementary file 1 [file biomedicines-13-02477-s001.zip › Figure S4.jpg]

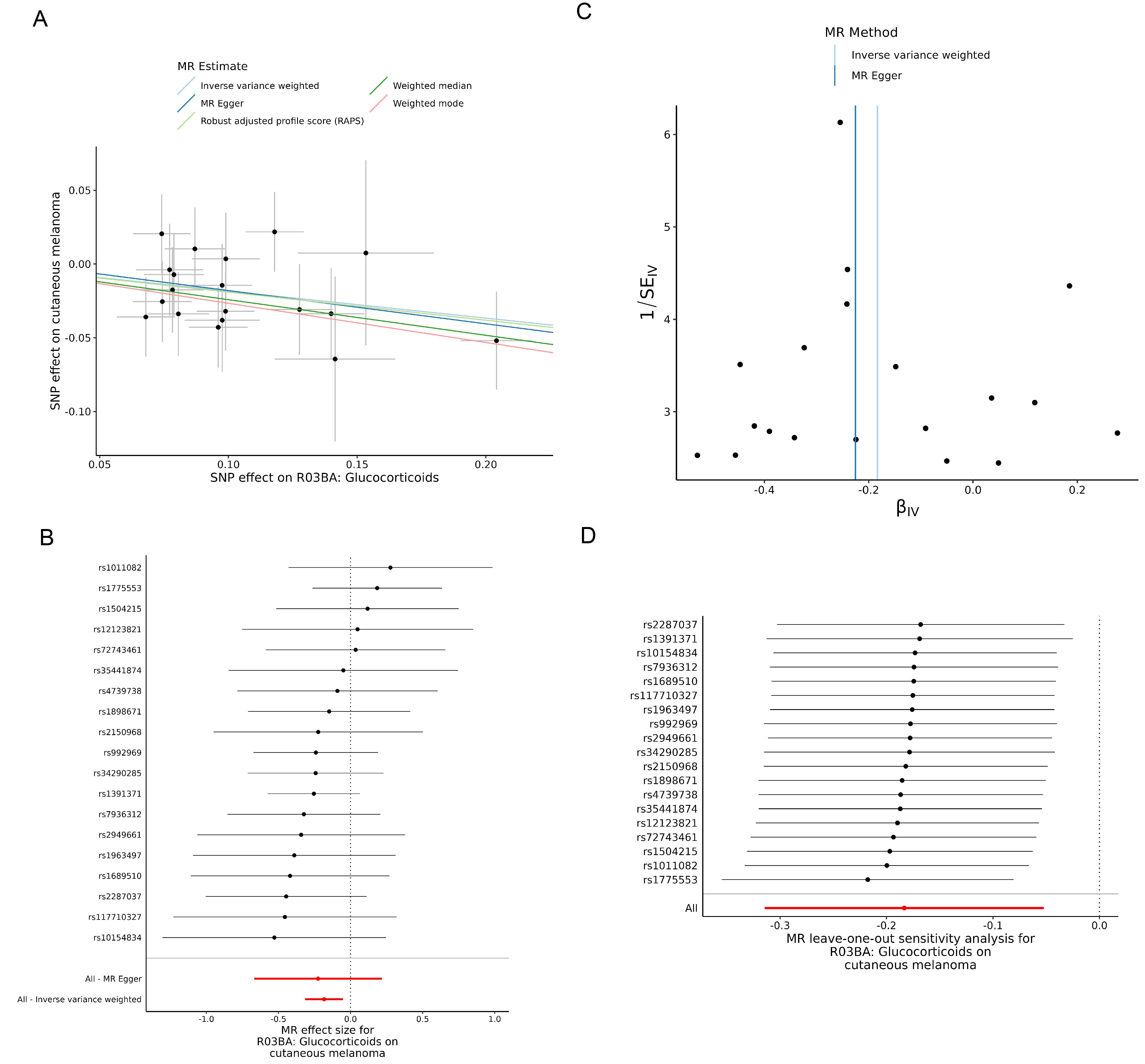

Supplement: Supplementary file 1 [file biomedicines-13-02477-s001.zip › Figure S5.jpg]

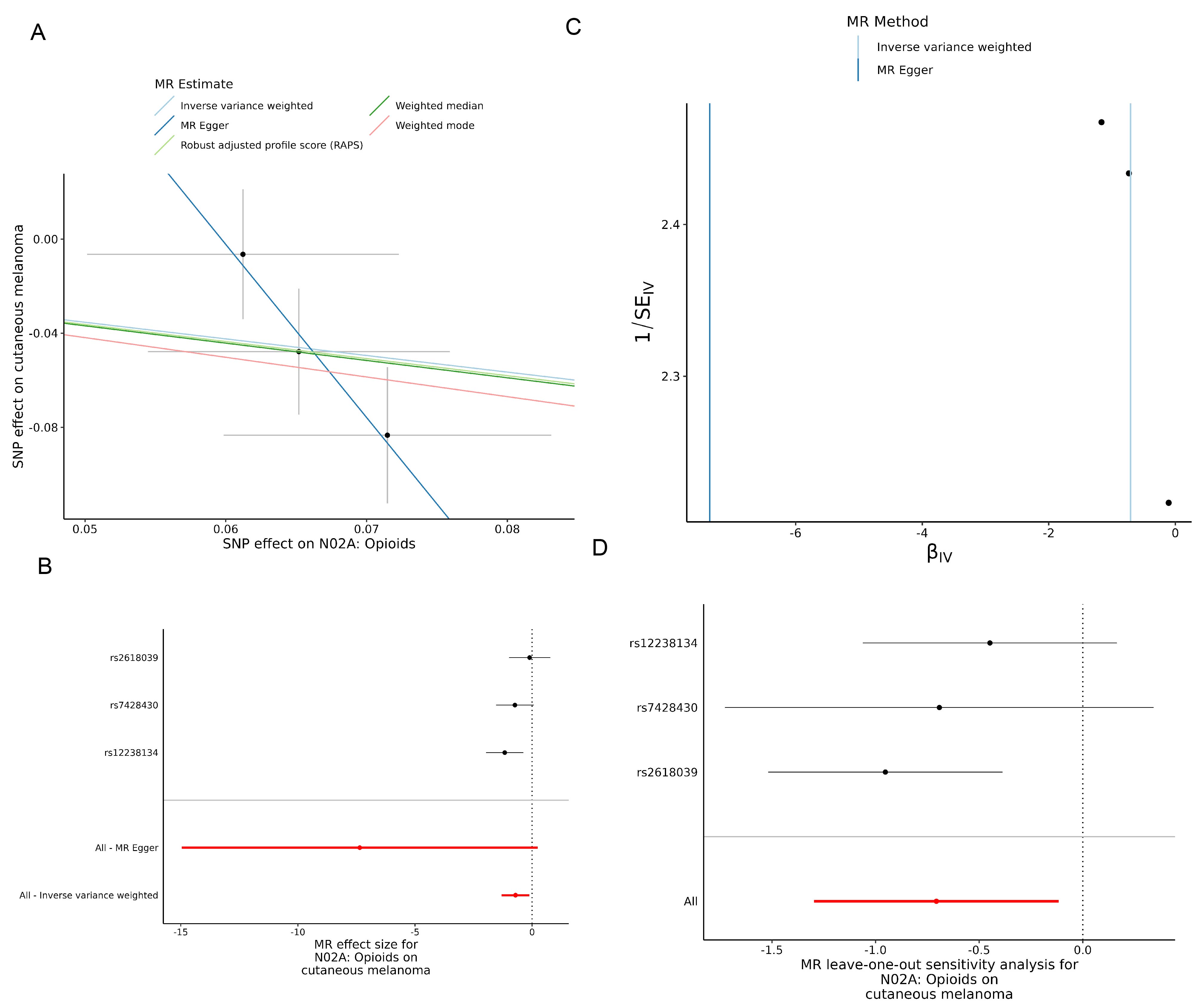

Supplement: Supplementary file 1 [file biomedicines-13-02477-s001.zip › Figure S6.jpg]
